# Can the performance of pyrethroid-chlorfenapyr nets be reduced when combined with pyrethroid-piperonyl butoxide (PBO) nets?

**DOI:** 10.1186/s12936-023-04648-6

**Published:** 2023-07-21

**Authors:** Thomas Syme, Judicaël Nounagnon, Boris N’dombidjé, Martial Gbegbo, Abel Agbevo, Juniace Ahoga, Corine Ngufor

**Affiliations:** 1 London School of Hygiene & Tropical Medicine, London, UK; 2 Centre de Recherche Entomologique de Cotonou, Cotonou, Benin; 3 Pan African Malaria Vector Research Consortium (PAMVERC), Cotonou, Benin

**Keywords:** Insecticide-treated nets, Chlorfenapyr, Piperonyl butoxide, Pyrethroid, Vector control, Anopheles gambiae, Mosquitoes, Malaria, Experimental huts, Pro-insecticide, Antagonism

## Abstract

**Background:**

Pyrethroid-chlorfenapyr (CFP) and pyrethroid-piperonyl butoxide (PBO) nets are being scaled across endemic countries to improve control of malaria transmitted by pyrethroid-resistant mosquitoes. CFP is a pro-insecticide requiring activation by mosquito cytochrome P450 monooxygenase enzymes (P450s) while PBO improves pyrethroid potency by inhibiting the action of these enzymes in pyrethroid-resistant mosquitoes. The inhibitory action of PBO against P450s may thus reduce the efficacy of pyrethroid-CFP nets when applied inside the same household as pyrethroid-PBO nets.

**Methods:**

Two experimental hut trials were performed to evaluate the entomological impact of two different types of pyrethroid-CFP ITN (Interceptor^®^ G2, PermaNet^®^ Dual) when applied alone and in combination with pyrethroid-PBO ITNs (DuraNet^®^ Plus, PermaNet^®^ 3.0) against a pyrethroid-resistant vector population in southern Benin. In both trials, all net types were tested as single and double net treatments. Bioassays were also performed to assess the resistance profile of the vector population at the hut site and investigate interactions between CFP and PBO.

**Results:**

The vector population was susceptible to CFP but exhibited a high intensity of pyrethroid resistance that was overcame by PBO pre-exposure. Vector mortality was significantly lower in huts with combinations of pyrethroid-CFP nets plus pyrethroid-PBO nets compared to huts with two pyrethroid-CFP nets (74% vs. 85% for Interceptor^®^ G2 and 57% vs. 83% for PermaNet^®^ Dual, p < 0.001). PBO pre-exposure reduced the toxicity of CFP in bottle bioassays suggesting this effect may be partly attributable to antagonism between CFP and PBO. Higher levels of vector mortality were observed in huts with net combinations that included pyrethroid-CFP nets compared to those that did not and highest mortality was achieved when pyrethroid-CFP nets were applied alone as two nets together (83–85%).

**Conclusions:**

This study shows evidence of a reduced performance of pyrethroid-CFP nets when combined with pyrethroid-PBO ITNs compared to when applied alone and higher efficacy with net combinations that included pyrethroid-CFP nets. These findings suggest that in similar contexts, prioritizing distribution of pyrethroid-CFP nets over other net types would maximize vector control impact.

**Supplementary Information:**

The online version contains supplementary material available at 10.1186/s12936-023-04648-6.

## Background

Insecticide-treated nets (ITNs) containing pyrethroid insecticides have been the primary malaria control intervention for the past two decades. Approximately 2.5 billion ITNs have been supplied to sub-Saharan Africa since 2004 [1] causing the proportion of the population sleeping under an ITN to increase from 4 to 47% [2]. The impact of this roll-out has been remarkable. Globally, an estimated 2 billion malaria cases and 6.2 million deaths were prevented between 2000 and 2021 and modelling analyses indicate that ITNs were the main driver of this benefit [2, 3]. These gains have, however, come at a cost: the accelerated evolution of pyrethroid resistance in malaria vector populations. Although pyrethroid ITNs still provide personal protection against malaria infection in areas where vectors exhibit pyrethroid resistance [4], modelling studies predict reduced epidemiological impact of ITNs at higher levels of resistance [5]. Pyrethroid resistance thus represents one of the most significant threats to sustaining malaria control progress.

In the last few years, a new generation of ITNs combining a pyrethroid with a second chemical has been developed to improve control of malaria transmitted by pyrethroid-resistant mosquitoes. The first novel ITN class contains the synergist piperonyl butoxide (PBO), which enhances the potency of pyrethroids by neutralizing detoxifying enzymes associated with pyrethroid resistance, notably cytochrome P450 monooxygenases (P450s) [6]. More recently, nets treated with chlorfenapyr (CFP), a pyrrole insecticide with a novel mode of action targeting cellular respiration, have also become available. Following demonstration of improved entomological impact in experimental hut trials [7, 8], a series of cluster-randomized controlled trials (cRCTs) were conducted to evaluate the public health value of these nets relative to pyrethroid-only ITNs and generate the evidence necessary to inform a World Health Organization (WHO) policy recommendation [9]. Pyrethroid-PBO ITNs received WHO endorsement [10] based on evidence of improved epidemiological impact from cRCTs in Uganda [11] and Tanzania [12]. A policy recommendation for pyrethroid-CFP ITNs was also recently issued [10] after a prototype ITN (Interceptor^®^ G2) was shown to reduce child malaria incidence by 46% and 44% respectively in parallel cRCTs in Benin [13] and Tanzania [14].

With fresh impetus from the Global Fund and other major malaria control donors to address insecticide resistance through accelerated introduction of new nets [15], pyrethroid-PBO and pyrethroid-CFP ITNs are already replacing traditional pyrethroid-only ITNs in endemic areas. Between 2019 and 2022, the proportion of pyrethroid-PBO ITNs of all nets delivered to sub-Saharan Africa rose from 8 to 51% [1], while ‘dual-active’ ITNs including pyrethroid-CFP ITNs are projected to comprise 56% of the African market by 2025 [16]. As the body of evidence demonstrating the effectiveness of pyrethroid-PBO and pyrethroid-CFP ITNs grows, a more extensive roll-out of these nets is expected in the coming years. There is, therefore, an increased need to fill information gaps surrounding optimal deployment of next-generation ITNs to maximize impact when scaled up for full operational use.

An example of an operational research question raised by national malaria control programmes (NMCPs) considering concurrent distribution of pyrethroid-CFP and pyrethroid-PBO ITNs was: would the effectiveness of pyrethroid-CFP ITNs be reduced if they were deployed in the same household as pyrethroid-PBO ITNs? This concern arose because PBO works by inhibiting mosquito P450 enzymes [6] while CFP is a pro-insecticide requiring activation by P450s [17]. It was thus hypothesized that the inhibitory action of PBO against P450s could reduce the efficacy of pyrethroid-CFP ITNs when they are used in the same household as pyrethroid-PBO ITNs. Several laboratory studies show that PBO pre-exposure reduces the acute toxicity of CFP against vector mosquitoes in direct response-to-exposure bioassays [18–22]. However, the interaction between these chemicals will be more complex when played out between different nets in a field setting. No published trials have investigated the impact of co-deploying different types of ITN. Field studies evaluating the impact of combining pyrethroid-CFP and pyrethroid-PBO ITNs inside the same household will, therefore, help determine whether potential antagonism between these net types represents an operational concern and guide optimal deployment strategy in areas where their coincident distribution is being considered.

In this study, two experimental hut trials were performed to evaluate the entomological impact of two types of pyrethroid-CFP ITN (Interceptor^®^ G2 and PermaNet^®^ Dual) when applied alone and in combination with pyrethroid-PBO ITNs against a pyrethroid-resistant vector population in southern Benin. In the first trial, the impact of combining Interceptor^®^ G2 (an alpha-cypermethrin-CFP net) was tested with DuraNet^®^ Plus (an alpha-cypermethrin-PBO net), while in the second, the impact of combining PermaNet^®^ Dual (a deltamethrin-CFP net) was tested with PermaNet^®^ 3.0 (a deltamethrin-PBO net). In both trials, all ITN types were tested as single and double net treatments. Laboratory bioassays were performed to assess the susceptibility of the vector population at the hut site to the insecticides used in the ITNs and investigate interactions between CFP and PBO. Net pieces cut from ITNs before and after the hut trials were also tested in tunnel tests and analysed for chemical content.

## Methods

### Susceptibility bioassays

WHO susceptibility bioassays [23] were performed during each trial using F1 progeny of *Anopheles gambiae *sensu lato* (s.l.)* collected from the experimental hut site to assess the susceptibility of the Covè vector population to the active ingredients in the ITNs. Mosquitoes were exposed in bottle bioassays to the discriminating concentrations (DCs) of alpha-cypermethrin (12.5 µg) and CFP (100 µg) and in tube tests to filter papers impregnated with the DC of deltamethrin (0.05%) to assess susceptibility to these insecticides. Further exposures were performed with various multiples of the DCs of alpha-cypermethrin and deltamethrin to assess pyrethroid resistance intensity. To assess synergism and the role of P450s in pyrethroid resistance, mosquitoes were also exposed to bottles coated with the DC of alpha-cypermethrin (12.5 µg) with pre-exposure to PBO (400 µg/bottle) and filter papers impregnated with the DC of deltamethrin (0.05%) with pre-exposure to PBO (4%/paper). To prepare test bottles, stock solutions were prepared for each insecticide and dose by dissolving technical grade insecticide in acetone. Bottles were then coated by introducing 1 ml of stock solution into bottles and rotating using a tube roller. Approximately 100 3–5 day-old mosquitoes were exposed to each insecticide and dose for 60 min in four batches of 25. Concurrent exposures were performed with PBO alone, acetone-coated bottles and silicone oil-impregnated papers as controls. Knockdown was recorded at the end of exposure, after which mosquitoes were transferred to untreated containers, provided access to 10% (w/v) glucose solution and held at 27 ± 2 °C and 75 ± 10% relative humidity (RH). Delayed mortality was recorded after 24 h for the alpha-cypermethrin and deltamethrin exposures and every 24 h up to 72 h for CFP.

### Experimental hut trials

The overall aim of this study was to evaluate the entomological impact of combining pyrethroid-CFP and pyrethroid-PBO ITNs inside the same household. Experimental hut trials are standardized simulations of human-occupied housing recommended by the WHO for the evaluation of indoor vector control interventions. Mosquitoes enter huts at night and interact ad libitum with the human host and vector control intervention(s) contained inside. In the morning on each day of the trial, mosquitoes are then collected from each hut and scored for entomological outcomes correlated with epidemiological impact [24], notably mortality and blood-feeding. Compared to other test designs, experimental huts provide a more realistic representation of a household setting with a compact design allowing for natural interactions of wild, free-flying mosquitoes with two nets.

### Study site and experimental huts

Both experimental hut trials were performed at CREC/LSHTM field station in Covè, southern Benin (7°14′N2°18′E). The huts are located in a vast area of rice irrigation that provides permanent and extensive mosquito breeding sites. *Anopheles coluzzii* and *An. gambiae *sensu stricto (*s.s.*) occur sympatrically with the former predominating particularly in the rainy season. The Covè vector population exhibits a high frequency (≥ 90%) and intensity (200-fold) of pyrethroid and organochlorine resistance driven by the presence of the knockdown resistance (*kdr*) L1014F mutation and overexpression of P450s [25]. It remains susceptible to other unrelated insecticides including CFP [26]. Experimental huts used were of standard West African design but with larger dimensions (15 m^3^) to accommodate two sleeping areas. They were constructed from concrete bricks with cement-plastered walls, a corrugated iron roof and a polyethylene ceiling. Mosquitoes entered via four window slits with a 1 cm opening positioned on two sides of the hut. A wooden-framed veranda projected from the rear wall of each hut to capture exiting mosquitoes. Huts were surrounded by a water-filled moat to preclude mosquito predators. Diagrams showing the design of the experimental huts and division of hut rooms into equally sized sleeping areas are provided as supplementary information (Additional file 1: Figs. S1, S2).

### Experimental hut treatments

Two experimental hut trials were performed to evaluate the entomological impact of different types of pyrethroid-CFP ITN and pyrethroid-PBO ITN applied alone and in combination inside the same household. Both trials consisted of two components. The first component compared the efficacy of each ITN type applied singly while the second compared different combinations of ITNs applied together inside the same experimental hut.

Trial 1 evaluated the impact of combining the alpha-cypermethrin-based ITNs; Interceptor^®^ G2 (BASF) and DuraNet^®^ Plus (Shobikaa Impex). Comparison was also made to a combination of Interceptor^®^ G2 and Interceptor^®^ (BASF), an alpha-cypermethrin based pyrethroid-only ITN, to control for confounding effects of the pyrethroid. The following treatments were tested in trial 1:

1. Untreated net (control I)
2. Interceptor^®^
3. DuraNet^®^ Plus
4. Interceptor^®^ G2
5. Untreated net + Untreated net (control II)
6. Interceptor^®^ + Interceptor^®^
7. DuraNet^®^ Plus + DuraNet^®^ Plus
8. Interceptor^®^ G2 + Interceptor^®^ G2
9. Interceptor^®^ G2 + Interceptor^®^
10. Interceptor^®^ G2 + DuraNet^®^ Plus

Trial 2 evaluated the impact of combining the deltamethrin-based ITNs; PermaNet^®^ Dual and PermaNet^®^ 3.0 (Vestergaard Sàrl). Interceptor^®^ G2 was also included as a single net treatment arm in trial 2 to compare its performance to PermaNet^®^ Dual. The following treatments were tested in trial 2:

1. Untreated net (control I)
2. PermaNet^®^ 3.0
3. Interceptor^®^ G2
4. PermaNet^®^ Dual
5. Untreated net + Untreated net (control II)
6. PermaNet^®^ 3.0 + PermaNet^®^ 3.0
7. PermaNet^®^ Dual + PermaNet^®^ Dual
8. PermaNet^®^ Dual + PermaNet^®^ 3.0

### ITN characteristics and preparation

- Interceptor^®^ is a WHO-prequalified pyrethroid-only ITN made of polyester filaments coated with 5 g/kg of alpha-cypermethrin.
- DuraNet^®^ Plus is a WHO-prequalified pyrethroid-PBO ITN made of polyethylene monofilament incorporated with 6 g/kg of alpha-cypermethrin and 2.2 g/kg of PBO.
- Interceptor^®^ G2 is a WHO-prequalified pyrethroid-CFP ITN made of polyester filaments coated with 2.4 g/kg of alpha-cypermethrin and 4.8 g/kg of CFP.
- PermaNet^®^ 3.0 is a WHO-prequalified pyrethroid-PBO ITN which consists of polyester side panels coated with 2.1 g/kg of deltamethrin and a polyethylene roof panel incorporated with 4 g/kg of deltamethrin and 25 g/kg of PBO.
- PermaNet^®^ Dual is a WHO-prequalified pyrethroid-CFP ITN made of polyester filaments coated with 2.1 g/kg of deltamethrin and 5 g/kg of CFP.

Six (6) replicate nets were selected for each net type per treatment arm for the experimental hut trials and rotated them within treatments daily. Bed nets were erected over sleeping areas inside huts by tying the corners of the roof panel to nails positioned on the uppermost sides of hut walls. In huts containing single nets, nets were hung in the centre of the room while in huts containing two nets, the hut room was divided vertically into two equally sized sleeping areas with nets hung on either side. All nets were given 6 holes each measuring 4 × 4 cm to mimic wear-and-tear from routine use.

### Treatment and sleeper rotation

In both trials, treatments were rotated between experimental huts weekly according to randomized Latin square designs (LSDs) to mitigate bias due to differences in positional attractiveness of experimental huts. For net combination arms, nets were also rotated between sleeping areas inside huts to reduce bias due to mosquito entry point preference. Human volunteers were recruited to sleep in experimental huts between 21:00 and 06:00 to attract wild, free-flying mosquitoes. Volunteers were randomly assigned to sleep alone in single net treatments or as pairs in double net treatments. Single sleepers and sleeper pairs were rotated according to separate LSDs to mitigate bias due to individual attractiveness to mosquitoes.

### Mosquito collections and processing

Each morning, volunteers collected mosquitoes from the different hut compartments (under the net, room, veranda) and deposited them in labelled plastic cups. Mosquito collections were then transferred to the field laboratory for morphological identification and scoring of immediate mortality and blood-feeding. Surviving, female *An. gambiae s.l.* were provided access to 10% (w/v) glucose solution and delayed mortality was recorded every 24 h up to 72 h after collection for all treatments. Mosquito collections were performed 6 days per week and on the 7th day, huts were cleaned to prevent contamination before the next rotation cycle. In both trials, mosquito collections continued for two full treatment rotations equating to 20 weeks for trial 1 between October, 2021 and March, 2022 and 16 weeks for trial 2 between May and October, 2022.

### Experimental hut trial outcome measures

The efficacy of the experimental hut treatments was expressed in terms of the following outcome measures:

1. **Hut entry**—number of mosquitoes collected in experimental huts
2. **Deterrence (%)**—reduction in the number of mosquitoes collected in the treated hut relative to the untreated control hut. Calculated as follows:

Where *Tu* is the number of mosquitoes collected in the untreated control hut and *Tt* is the number of mosquitoes collected in the treated hut.

3. **Exophily (%)**—exiting rates due to potential irritant effects of a treatment expressed as the proportion of mosquitoes collected in the veranda
4. **Inside net (%)**—proportion of mosquitoes collected inside the net
5. **Blood-feeding (%)**—proportion of blood-fed mosquitoes
6. **Blood-feeding inhibition (%)**—proportional reduction in blood-feeding in the treated hut relative to the untreated control hut. Calculated as follows:

Where *Bfu* is the proportion of blood-fed mosquitoes in the untreated control hut and *Bft* is the proportion of blood-fed mosquitoes in the treated hut.

7. **Personal protection (%)**—reduction in the number of blood-fed mosquitoes in the treated hut relative to the untreated control hut. Calculated as follows:

Where *Bu* is the number of blood-fed mosquitoes in the untreated control hut and *Bt* is the number of blood-fed mosquitoes in the treated hut.

8. **Delayed mortality (%)**—proportion of dead mosquitoes observed every 24 h up to 72 h after collection
9. **Overall killing effect (%)**—number of mosquitoes killed in the treated hut relative to the number collected in the untreated control hut. Calculated as follows:

Where *Kt* is the number of dead mosquitoes in the treated hut, *Ku* is the number of dead mosquitoes in the untreated control hut and *Tu* is the number of mosquitoes collected in the untreated control hut.

### Chlorfenapyr and PBO interaction bioassays

The entomological performance of pyrethroid-CFP ITNs when combined with pyrethroid-PBO ITNs inside experimental huts may also be influenced by the behaviour of the wild vector population. Bottle bioassays were thus performed to assess the impact of PBO pre-exposure on CFP toxicity. By eliminating confounding effects associated with mosquito behaviour, these bioassays were used to investigate interactions between CFP and PBO and thus help explain findings from the experimental hut trials. The bioassays were performed with the pyrethroid-resistant *An. gambiae s.l.* Covè strain, which are F1 progeny of wild mosquitoes collected from the experimental hut site. Test bottles were coated with the DC of PBO (400 µg), and 0.25x (25 µg), 0.5 (50 µg), 0.75x (75 µg) and 1x (100 µg) the DC of CFP as previously described. Cohorts of approximately 150, 3–5 day-old mosquitoes were subsequently exposed to each dose for 60 min with and without pre-exposure to the discriminating dose of PBO (400 µg) in 6 replicates of 25. Mosquitoes were then transferred to untreated containers with access to 10% (w/v) glucose solution and held at 27 ± 2 °C and 75 ± 10% RH. Knockdown was recorded at the end of exposure and delayed mortality every 24 h up to 72 h.

### Preparation of net pieces for bioassays and chemical analysis

In each trial, 5 net pieces (one from each panel) measuring 30 × 30 cm were cut from 1 new, unused net and 2 nets used in experimental huts for each ITN type for laboratory bioassays and chemical analysis. Because of the mosaic design of PermaNet^®^ 3.0, two additional net pieces were cut from the roof panel to provide 7 pieces in total and ensure appropriate representation of PBO-incorporated pieces as per WHO specifications [27]. Net pieces were wrapped in labelled aluminium foil and stored at 30 ± 2 °C before and during use in supplementary tunnel tests. Following use in tunnel tests, net pieces were stored at 4 ± 2°C before being sent for chemical analysis.

### Supplementary tunnel tests

The inappropriateness of cone bioassays for evaluating the efficacy of pyrethroid-CFP ITNs is well-documented [28]. Tunnel tests were thus performed with two net pieces randomly selected from those cut from ITNs before and after the hut trials to provide supplementary ITN efficacy data. The tunnel tests were performed with the susceptible *An. gambiae s.s.* Kisumu strain to assess the pyrethroid component of the ITNs and the pyrethroid-resistant *An. gambiae s.l.* Covè strain to assess the additional impact of the CFP and PBO components.

- *An. gambiae s.s.* Kisumu strain is an insecticide-susceptible reference strain originated from Kisumu, western Kenya.
- *An. gambiae s.l.* Covè strain are F1 progeny of mosquitoes collected from the experimental hut site in Covè, southern Benin. This strain is highly resistant to pyrethroids and organochlorines but susceptible to other insecticide classes including CFP. Resistance is mediated by the *kdr* L1014F mutation and overexpression of P450s [25].

Tunnel tests are an experimental chamber used to evaluate the efficacy of ITNs which simulate the behavioural interactions that occur between free-flying mosquitoes and nets during host-seeking. The design consists of a square glass tunnel divided at one third its length by a wooden frame fitted with a net piece. In the shorter section of the tunnel, a guinea pig bait was held in an open-meshed cage while in the longer section, approximately 100, 5–8 day old mosquitoes were released at dusk and left overnight. Net pieces were given 9 holes measuring 1 cm in diameter to facilitate entry into the baited chamber. In the morning, mosquitoes were collected from the tunnel and scored for immediate mortality and blood-feeding. Live mosquitoes were transferred to labelled plastic cups, provided access to 10% (w/v) glucose solution and held at 27 ± 2 °C and 75 ± 10% RH. Delayed mortality was recorded every 24 h up to 72 after exposure. Mosquitoes were concurrently exposed to untreated net pieces as a negative control.

### Chemical analysis of ITNs

All net pieces cut from ITNs of each type before and after the experimental hut trials were sent to accredited analytical laboratories to confirm that the chemical contents of all ITNs fell within WHO tolerance thresholds (± 25%) before use in experimental huts and assess how this changed after the hut trial. The methods used for analysis of chemical content have been described in a previous publication [29]. The results confirmed that the chemical contents of the ITNs were within WHO tolerance thresholds (± 25%) before the hut trials except for the PBO content in DuraNet® Plus pieces (+ 36%). Chemical content of all active ingredients changed slightly after the hut trials but remained within the ± 25% threshold. Detailed chemical analysis results are provided as supplementary information (Additional file 1: Table S1).

### Data analysis

For experimental hut trial data, differences between treatments for proportional binary outcomes (mortality, blood-feeding, exophily) were analysed using blocked logistic regression and differences between count outcomes (entry) using negative binomial regression. Separate models were fitted for each outcome and adjusted for variation associated with the different huts, sleepers/sleeper pairs and weeks of the trial. These analyses were performed in Stata version 17. Insecticide resistance bioassay data was interpreted according to WHO criteria [23] while interaction bioassay and tunnel test results were plotted on graphs to visualize differences between treatments for key outcomes.

### Ethical considerations

Approval for the conduct of the trials was received from the Research Ethics Committees of the Benin Ministry of Health (N°133, 17/11/2021) and the London School of Hygiene & Tropical Medicine (LSHTM) (Ref: 26429). All participants provided written informed consent prior to the study onset. Participants were offered a free course of chemoprophylaxis spanning the duration of the study and up to 4 weeks following its completion to mitigate malaria risk. A stand-by nurse was also available to assess any febrile illness or adverse reactions to the test items. LSHTM Animal Welfare Ethics Review Board issued approval for the use of guinea pigs for mosquito blood-feeding and tunnel tests (Ref: 2020-01A). Guinea pig colonies were maintained according to protocols developed in line with international guidelines governing the use of animals for scientific research.

## Results

### Resistance bioassay results

**Fig. 1:**
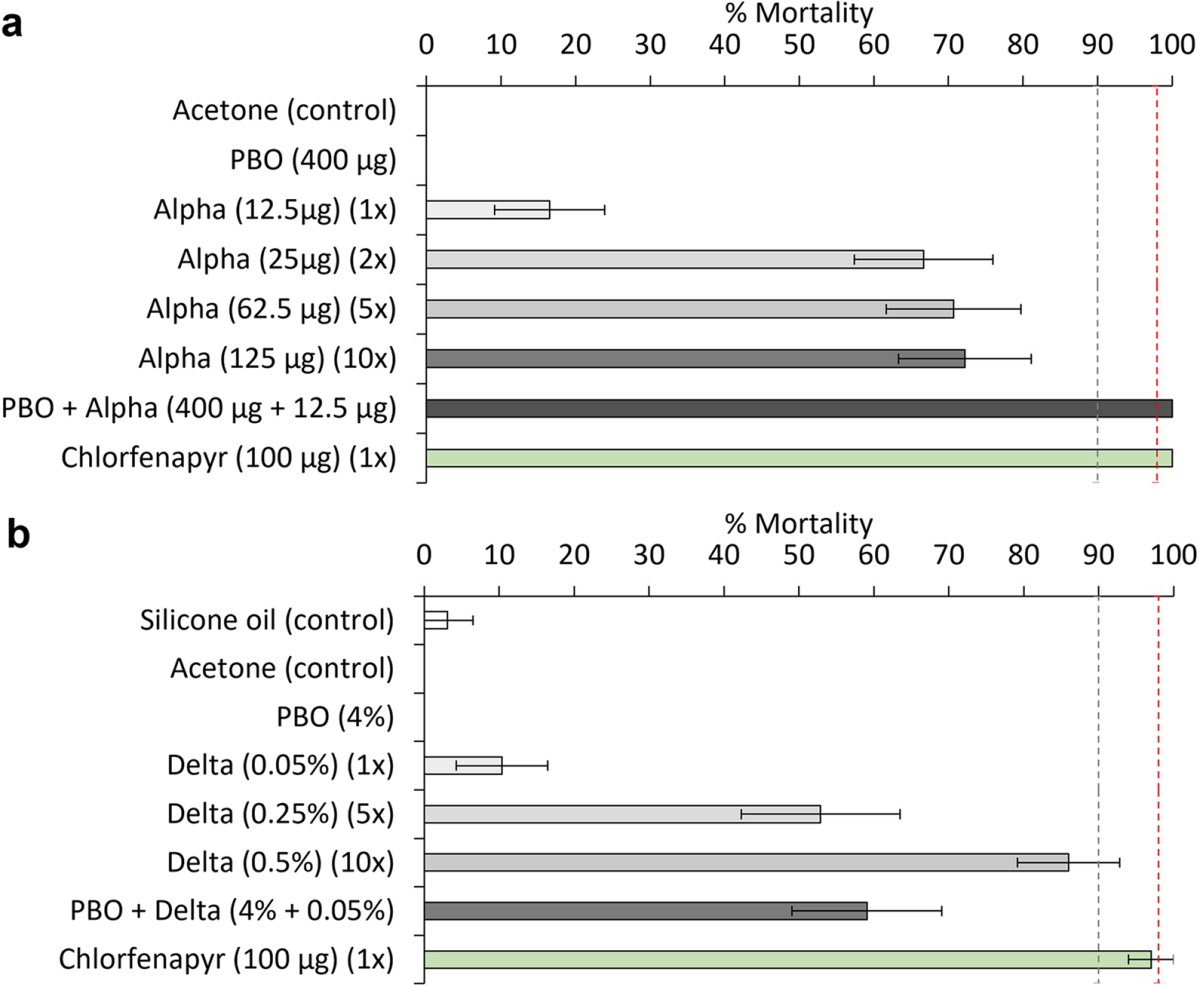
Mortality of F1 progeny of *Anopheles gambiae s.l.* collected from the experimental hut site in Covè in World Health Organization tube tests and bottle bioassays. Panel a presents results from trial 1 and panel b presents results from trial 2. In trial 1, all insecticides were tested in bottle bioassays while in trial 2, only chlorfenapyr was tested in bottle bioassays. Red dashed line represents standard 98% susceptibility cut-off while grey dashed line represents provisional 90% susceptibility cut-off used to confirm chlorfenapyr resistance. Error bars represent 95% CIs. Panel a presents results with single nets and panel b presents results with double net combinations

Low mosquito mortality was observed following exposure to the DCs of alpha-cypermethrin (12.5 µg) (17%) and deltamethrin (0.05%) (10%), confirming the high frequency of pyrethroid resistance in the Covè vector population during both trials (Fig. 1). The mortality response improved with 2x (67%), 5x (71%) and 10x (72%) the DC of alpha-cypermethrin and 5x (53%) and 10x (86%) the DC of deltamethrin. Mortality failed to exceed the 98% threshold with either insecticide at any dose indicating the presence of high-intensity pyrethroid resistance during both trials. Pre-exposure to PBO fully restored susceptibility to alpha-cypermethrin (100% mortality) and partially restored susceptibility to deltamethrin (59% mortality) thus implicating the involvement of P450s in pyrethroid resistance. No mortality was recorded in mosquitoes exposed to PBO alone. CFP induced very high mortality during both trial 1 (97%) and trial 2 (100%), demonstrating the susceptibility of the vector population to this insecticide. Mortality was negligible with the acetone (0%) and silicone oil controls (3%). Detailed resistance bioassay results are provided as supplementary information (Additional file 1: Tables S2, S3).

### Experimental hut results

#### Experimental hut results with single nets

**Fig. 2:**
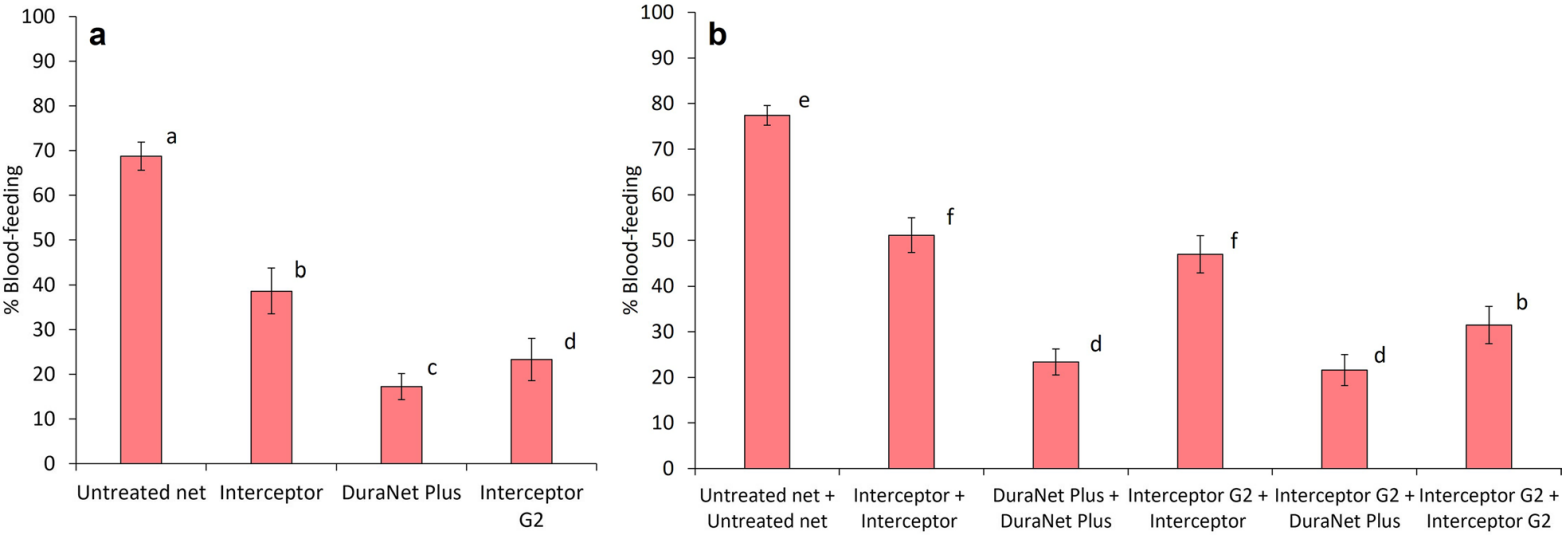
Blood-feeding rates of wild, pyrethroid-resistant *Anopheles gambiae s.l.* collected in experimental huts containing alpha-cypermethrin-chlorfenapyr nets (Interceptor^®^ G2) and alpha-cypermethrin-piperonyl butoxide nets (DuraNet^®^ Plus) applied alone and in combination in Covè, Benin (Trial 1). Panel a presents results with single net treatments and panel b presents results with double net combinations. Bars bearing the same letter do not differ significantly at 5% level (p > 0.05) according to logistic regression analysis. Error bars represent 95% confidence intervals

**Fig. 3:**
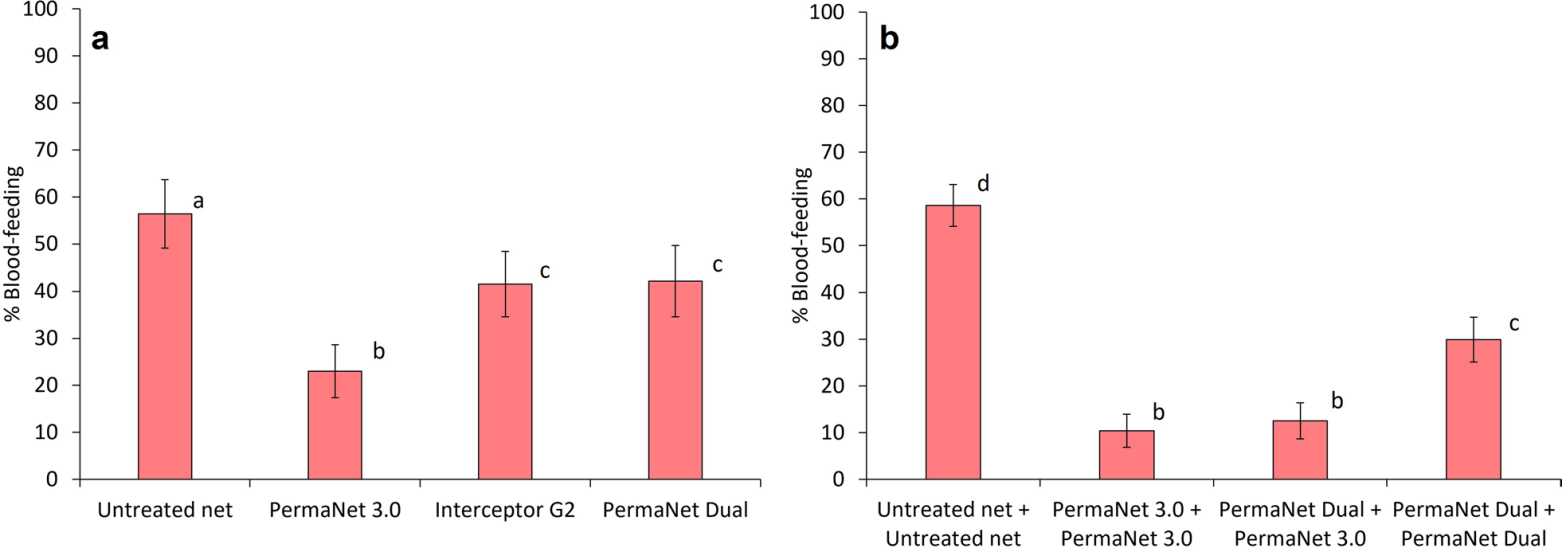
Blood-feeding rates of wild, pyrethroid-resistant *Anopheles gambiae s.l.* collected in experimental huts containing deltamethrin-chlorfenapyr nets (PermaNet^®^ Dual) and deltamethrin-piperonyl butoxide nets (PermaNet^®^ 3.0) applied alone and in combination in Covè, Benin (Trial 2). Panel a presents results with single net treatments and panel b presents results with double net combinations. Bars bearing the same letter do not differ significantly at 5% level (p > 0.05) according to logistic regression analysis. Error bars represent 95% confidence intervals

**Fig. 4:**
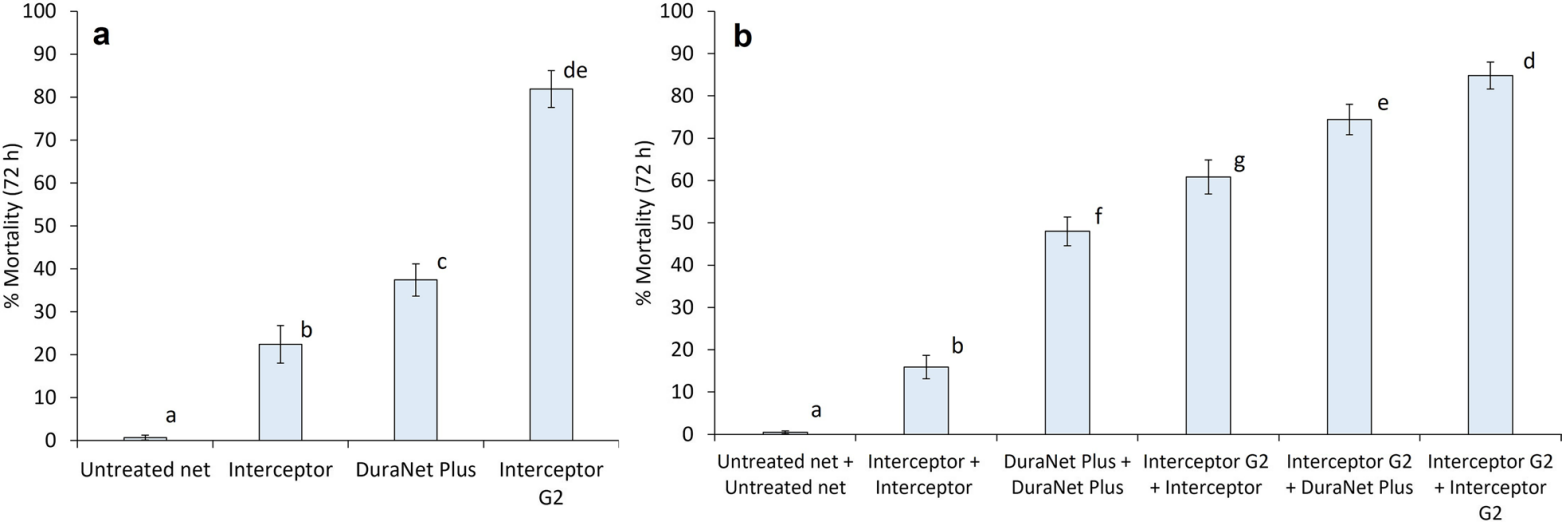
Mortality rates of wild, pyrethroid-resistant *Anopheles gambiae s.l.* collected in experimental huts containing alpha-cypermethrin-chlorfenapyr nets (Interceptor^®^ G2) and alpha-cypermethrin-piperonyl butoxide nets (DuraNet^®^ Plus) applied alone and in combination in Covè, Benin (Trial 1). Panel a presents results with single net treatments and panel b presents results with double net combinations. Bars bearing the same letter do not differ significantly at 5% level (p > 0.05) according to logistic regression analysis. Error bars represent 95% confidence intervals

**Fig. 5:**
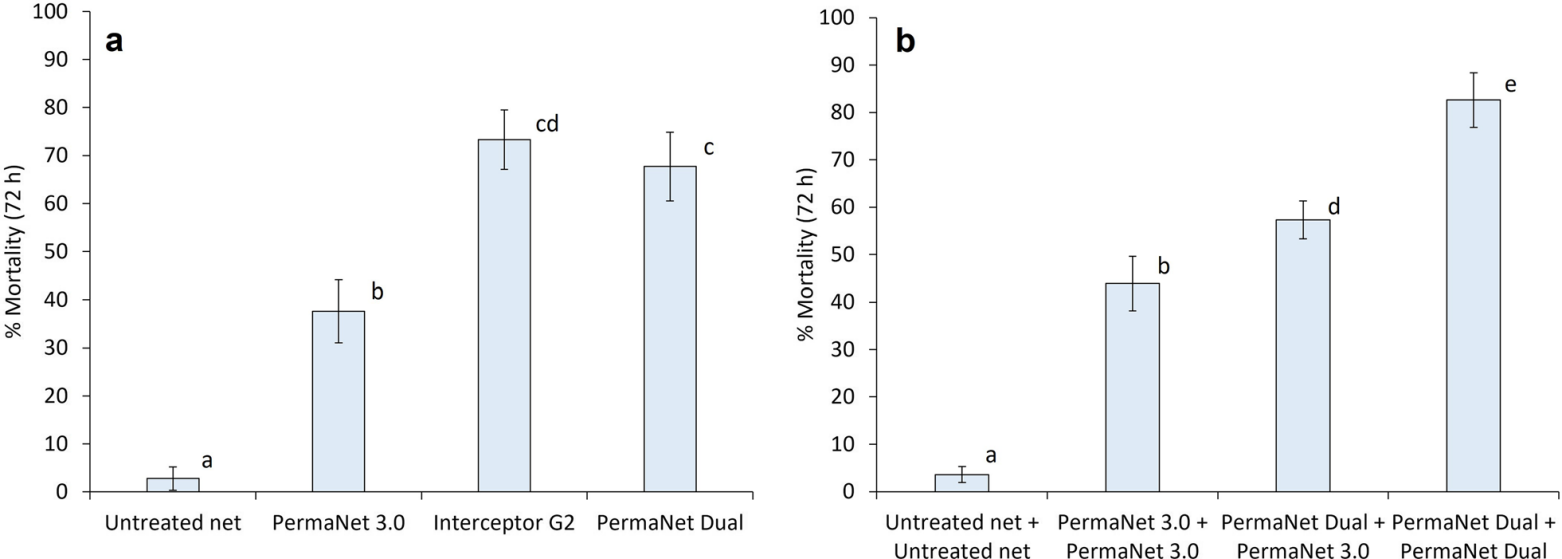
Mortality rates of wild, pyrethroid-resistant *Anopheles gambiae s.l.* collected in experimental huts containing deltamethrin-chlorfenapyr nets (PermaNet^®^ Dual) and deltamethrin-piperonyl butoxide nets (PermaNet^®^ 3.0) applied alone and in combination in Covè, Benin (Trial 2). Panel a presents results with single net treatments and panel b presents results with double net combinations. Bars bearing the same letter do not differ significantly at 5% level (p > 0.05) according to logistic regression analysis. Error bars represent 95% confidence intervals

A total of 2891 mosquitoes were collected in single net treatments across both trials. Significant deterrent effects were observed with all ITNs in trial 1, but not trial 2 (Table 1). In both trials, exiting was significantly higher with all ITNs compared to the untreated control nets (p < 0.001) and the highest exophily was induced by the pyrethroid-PBO ITNs (DuraNet^®^ Plus: 74%, PermaNet^®^ 3.0: 72%). Pyrethroid-PBO ITNs provided superior levels of blood-feeding inhibition than pyrethroid-CFP ITNs (Trial 1: 75% with DuraNet^®^ Plus vs. 66% with Interceptor^®^ G2, p < 0.001, Trial 2: 59% with PermaNet^®^ 3.0 vs. 26% with Interceptor^®^ G2, p < 0.001 and 25% with PermaNet^®^ Dual, p < 0.001) (Figs. 2, 3, Table 2). Despite this, blood-feeding was lower with Interceptor^®^ G2 than with Interceptor^®^ in trial 1 (23% vs. 39%, p < 0.001), showing that pyrethroid-CFP ITNs were still superior to pyrethroid-only ITNs for blood-feeding prevention. Pyrethroid-CFP ITNs induced superior mortality rates of pyrethroid-resistant *An. gambiae s.l.* compared to pyrethroid-PBO ITNs across both trials (Trial 1: 82% with Interceptor^®^ G2 vs. 37% with DuraNet^®^ Plus, p < 0.001, Trial 2: 73% with Interceptor^®^ G2 and 68% with PermaNet^®^ Dual vs. 38% with PermaNet^®^ 3.0, p < 0.001) (Figs. 4, 5, Table 3). Nevertheless, mortality was significantly higher with DuraNet^®^ Plus compared to Interceptor^®^ in trial 1 (37% vs. 22%, p < 0.001) showing that pyrethroid-PBO ITNs were still superior to pyrethroid-only ITNs in terms of inducing vector mortality. Similar levels of vector mortality were observed between both types of pyrethroid-CFP ITN in trial 2 (73% with Interceptor^®^ G2 vs. 68% with PermaNet^®^ Dual, p = 0.386).

**Table 1:** Entry and exiting results of wild, pyrethroid-resistant *Anopheles gambiae s.l.* collected in experimental huts containing pyrethroid-chlorfenapyr nets and pyrethroid-piperonyl butoxide nets applied alone and in combination in Covè, Benin

Trial component	Treatment	N^*^	% Deterrence	N exiting	% Exophily^*^	95% CIs
Trial 1^*^						
Single nets	Untreated net	835^ab^	–	304	36.4^a^	33.1–39.7
	Interceptor	352^c^	57.8	230	65.3^bcd^	60.3– 70.3
	DuraNet Plus	644^de^	22.9	478	74.2^e^	70.8–77.6
	Interceptor G2	309^c^	63.0	223	72.2^ef^	67.2–77.2
Double net combinations	Untreated net + Untreated net	1423^f^	–	624	43.9^g^	41.3–46.5
	Interceptor + Interceptor	659^ad^	53.7	427	64.8^b^	61.2–68.4
	DuraNet Plus + DuraNet Plus	836^a^	41.3	603	72.1^cef^	69.1–75.1
	Interceptor G2 + Interceptor G2	495^be^	65.2	328	66.3^bf^	62.1–70.5
	Interceptor G2 + Interceptor	569^be^	60.0	366	64.3^b^	60.4–68.2
	Interceptor G2 + DuraNet Plus	574^be^	59.7	413	72.0^def^	68.3–75.7
Trial 2^*^						
Single nets	Untreated net	179^vw^	–	84	46.9^w^	39.6–54.2
	PermaNet 3.0	213^vwy^	− 19.0	154	72.3^xy^	66.3–78.3
	Interceptor G2	195^vw^	− 8.9	122	62.6^z^	55.8–69.4
	PermaNet Dual	164^w^	8.4	104	63.4^z^	56.0–70.8
Double net combinations	Untreated net + Untreated net	466^z^	–	202	43.3^w^	38.8–47.8
	PermaNet 3.0 + PermaNet 3.0	289^vwy^	38.0	231	79.9^x^	75.3–84.5
	PermaNet Dual + PermaNet Dual	351^xz^	24.7	237	67.5^yz^	62.6–72.4
	PermaNet Dual + PermaNet 3.0	281^y^	39.7	228	81.1^x^	76.5–85.7

^*^Results are presented separately for trials involving alpha-cypermethrin-based nets (trial 1) and deltamethrin-based nets (trial 2)

^*^For each trial, values in the same column do not differ at the 5% level (p > 0.05) according to regression analysis

**Table 2:** Blood-feeding results of wild, pyrethroid-resistant *Anopheles gambiae s.l.* collected in experimental huts with pyrethroid-chlorfenapyr nets and pyrethroid-piperonyl butoxide nets applied alone and in combination in Covè, Benin

Trial component	Treatment	N^*^	N blood-fed	% Blood-feeding^*^	95% CIs	% Blood-feeding inhibition	% Personal protection
Trial 1^*^							
Single nets	Untreated net	835^ab^	574	68.7^a^	65.6–71.8	–	–
	Interceptor	352^c^	136	38.6^b^	33.5–43.7	43.8	76.3
	DuraNet Plus	644^de^	111	17.2^c^	14.3–20.1	75.0	80.7
	Interceptor G2	309^c^	72	23.3^d^	18.6–28.0	66.1	87.5
Double net combinations	Untreated net + Untreated net	1423^f^	1101	77.4^e^	75.2–79.6	–	–
	Interceptor + Interceptor	659^ad^	337	51.1^f^	47.3–54.9	34.0	69.4
	DuraNet Plus + DuraNet Plus	836^a^	196	23.4^d^	20.5–26.3	69.8	82.2
	Interceptor G2 + Interceptor G2	495^be^	156	31.5^b^	27.4–35.6	59.3	85.8
	Interceptor G2 + Interceptor	569^be^	267	46.9^f^	42.8–51.0	39.4	75.7
	Interceptor G2 + DuraNet Plus	574^be^	124	21.6^d^	18.2–25.0	72.1	88.7
Trial 2^*^							
Single nets	Untreated net	179^vw^	101	56.4^w^	49.1–63.7	–	–
	PermaNet 3.0	213^vwy^	49	23.0^x^	17.4–28.7	59.2	51.5
	Interceptor G2	195^vw^	81	41.5^y^	34.6–48.4	26.4	19.8
	PermaNet Dual	164^w^	69	42.1^y^	34.5–49.7	25.4	31.7
Double net combinations	Untreated net + Untreated net	466^z^	273	58.6^z^	54.1–63.1	–	–
	PermaNet 3.0 + PermaNet 3.0	289^vwy^	30	10.4^x^	6.9–13.9	82.3	89.0
	PermaNet Dual + PermaNet Dual	351^xz^	105	29.9^y^	25.1–34.7	49.0	61.5
	PermaNet Dual + PermaNet 3.0	281^y^	35	12.5^x^	8.6–16.4	78.7	87.2

^*^Results are presented separately for trials involving alpha-cypermethrin-based nets (trial 1) and deltamethrin-based nets (trial 2)

^*^For each trial, values in the same column do not differ at the 5% level (p > 0.05) according to logistic regression analysis

**Table 3:** Mortality results of wild, pyrethroid-resistant *Anopheles gambiae s.l.* collected in experimental huts with pyrethroid-chlorfenapyr nets and pyrethroid-piperonyl butoxide nets applied alone and in combination in Covè, Benin

Trial component	Treatment	N^*^	N dead 72 h	% 72 h mortality*	95% CIs	% Overall killing effect
Trial 1^*^						
Single nets	Untreated net	835^ab^	6	0.7^a^	0.1–1.3	–
	Interceptor	352^c^	79	22.4^b^	18.0–26.8	8.7
	DuraNet Plus	644^de^	241	37.4^c^	33.7–41.1	28.1
	Interceptor G2	309^c^	253	81.9^de^	77.6–86.2	29.6
Double net combinations	Untreated net + Untreated net	1423^f^	7	0.5^a^	0.1–0.9	–
	Interceptor + Interceptor	659^ad^	105	15.9^b^	13.1–18.7	6.9
	DuraNet Plus + DuraNet Plus	836^a^	401	48.0^f^	44.6–51.4	27.7
	Interceptor G2 + Interceptor G2	495^be^	420	84.8^d^	81.6–88.0	29.0
	Interceptor G2 + Interceptor	569^be^	346	60.8^g^	56.8–64.8	23.8
	Interceptor G2 + DuraNet Plus	574^be^	427	74.4^e^	70.8–78.0	29.5
Trial 2^*^						
Single nets	Untreated net	179^vw^	5	2.8^v^	0.4–5.2	–
	PermaNet 3.0	213^vwy^	80	37.6^w^	31.1–44.1	41.9
	Interceptor G2	195^vw^	143	73.3^xy^	67.1–79.5	77.1
	PermaNet Dual	164^w^	111	67.7^x^	60.5–74.9	59.2
Double net combinations	Untreated net + Untreated net	466^z^	17	3.6^v^	1.9–5.3	–
	PermaNet 3.0 + PermaNet 3.0	289^vwy^	127	43.9^w^	38.2–49.6	23.6
	PermaNet Dual + PermaNet Dual	351^xz^	290	82.6^y^	78.6–86.6	58.6
	PermaNet Dual + PermaNet 3.0	281^y^	161	57.3^z^	51.5–63.1	30.9

^*^Results are presented separately for trials involving containing alpha-cypermethrin-based nets (trial 1) and deltamethrin-based nets (trial 2)

^*^For each trial, values in the same column do not differ at the 5% level (p > 0.05) according to logistic regression analysis

#### Experimental hut results with net combinations

##### Entry and exiting of wild malaria vector mosquitoes with net combinations

A total of 5,943 mosquitoes were collected in double net combination treatments across both trials. All ITN combinations induced significant deterrence relative to two untreated control nets except for the combination of two PermaNet^®^ Dual in trial 2 (25%, p = 0.08) (Table 1). Mosquito entry was also generally higher with double net combinations relative to the corresponding ITN types applied alone. In both trials, all ITN combinations induced significant exiting relative to two untreated control nets (p ≤ 0.001) and exiting was higher with combinations that included pyrethroid-PBO ITNs compared to those that did not. In trial 1, higher exiting was observed with Interceptor^®^ G2 plus DuraNet^®^ Plus (72%) compared to Interceptor^®^ G2 plus Interceptor^®^ (64%, p = 0.008) and two Interceptor^®^ G2 (66%, p = 0.066) although the latter comparison was not statistically significant. In trial 2 however, two PermaNet^®^ 3.0 and PermaNet^®^ Dual plus PermaNet^®^ 3.0 induced significantly stronger exiting responses compared to two PermaNet^®^ Dual (80% and 81% vs. 68%, p < 0.001) (Fig. 1).

##### Blood-feeding rates of wild malaria vector mosquitoes with net combinations

All ITN combinations significantly reduced blood-feeding relative to two untreated control nets (p < 0.001) (Table 2). In both trials, superior blood-feeding protection was observed with combinations which included pyrethroid-PBO ITNs. In trial 1, the combination of two DuraNet^®^ Plus and Interceptor^®^ G2 plus DuraNet^®^ Plus provided significantly lower blood-feeding than two Interceptor^®^ G2 (23% and 22% vs. 32%, p < 0.001) (Fig. 2). Similarly in trial 2, blood-feeding was significantly lower with the combination of two PermaNet^®^ 3.0 (10%) and PermaNet^®^ Dual plus PermaNet^®^ 3.0 (13%) compared to two PermaNet^®^ Dual (30%, p < 0.001) (Fig. 3). These comparisons show that the lower blood-feeding in these combinations was attributable to the pyrethroid-PBO ITNs and confirm the superior personal protection afforded by this ITN class. Despite this, lower blood-feeding was recorded with two Interceptor^®^ G2 (32%) compared to Interceptor^®^ G2 plus Interceptor^®^ (47%, p < 0.001) and two Interceptor^®^ (51%, p < 0.001) in trial 1 thus reaffirming that pyrethroid-CFP ITNs still provided superior blood-feeding protection than pyrethroid-only ITNs.

##### Mortality rates of wild malaria vector mosquitoes with net combinations

In both trials, the highest mortality rates of any treatment were achieved with the combination of two pyrethroid-CFP ITNs (85% with two Interceptor^®^ G2 in trial 1, 83% with two PermaNet^®^ Dual in trial 2) (Table 3). Mortality was reduced when combining pyrethroid-CFP ITNs with other ITN types compared to when two pyrethroid-CFP ITNs were applied together. In trial 1, mortality was lower with the combination of Interceptor^®^ G2 plus DuraNet^®^ Plus (74%) and Interceptor^®^ G2 plus Interceptor^®^ (61%) compared to two Interceptor^®^ G2 (85%, p < 0.001). Mortality was also lower with Interceptor^®^ G2 plus DuraNet^®^ Plus (74%) compared to Interceptor^®^ G2 alone (82%) although this difference was not statistically significant (p = 0.204) (Fig. 4). A similar trend was observed in trial 2 where mortality was significantly reduced with PermaNet^®^ Dual plus PermaNet^®^ 3.0 (57%) relative to two PermaNet^®^ Dual (83%, p < 0.001) and PermaNet^®^ Dual applied alone (68%, p < 0.001) (Fig. 5). Despite the reduced mortality with pyrethroid-CFP ITNs when combined with pyrethroid-PBO ITNs, the combination of Interceptor^®^ G2 plus DuraNet^®^ Plus (74%) was still superior to Interceptor^®^ G2 plus Interceptor^®^ (61%, p < 0.001) in trial 1. Mortality was also higher in huts with ITN combinations containing pyrethroid-CFP ITNs compared to combinations that did not, further demonstrating the superior killing effect of pyrethroid-CFP ITNs. Interceptor^®^ G2 plus DuraNet^®^ Plus (74%) and Interceptor^®^ G2 plus Interceptor^®^ (61%) induced higher mortality than two DuraNet^®^ Plus (48%, p < 0.001) and two Interceptor^®^ (16%, p < 0.001) in trial 1. Similarly in trial 2, mortality was higher with PermaNet^®^ Dual plus PermaNet^®^ 3.0 (57%) compared to two PermaNet^®^ 3.0 (44%, p < 0.001).

##### Chlorfenapyr and PBO interaction bioassay results

**Fig. 6:**
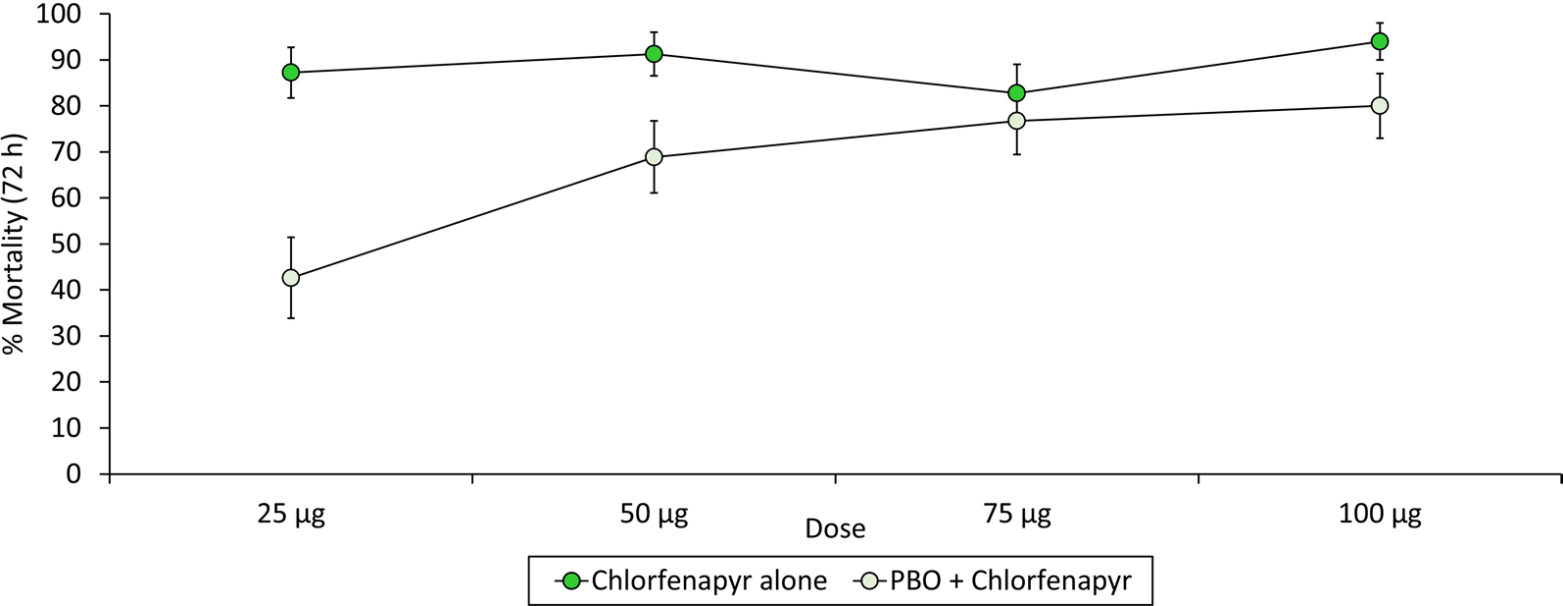
Mortality of pyrethroid-resistant *Anopheles gambiae s.l.* Covè strain exposed to bottles coated with chlorfenapyr with and without pre-exposure to piperonyl butoxide. Cohorts of approximately 150 mosquitoes were exposed to each treatment arm in six batches of 20–25

Mortality of the pyrethroid-resistant *An gambiae s.l.* Covè strain exposed to bottles coated with CFP alone was high (> 80%) at all doses tested. Pre-exposure to PBO reduced the mortality response to CFP at all doses tested; 25 μg (87% vs. 43%), 50 μg (91% vs. 69%), 75 μg (83% vs. 77%) and 100 μg (94% vs. 80%) (Fig. 6). The interaction bioassays therefore showed an antagonistic effect of PBO on the toxicity of chlorfenapyr. PBO alone induced negligible mortality (3%), which was similar to the acetone control (2%). Detailed CFP and PBO interaction bioassays results are provided as supplementary information (Additional file 1: Table S4).

##### Supplementary tunnel test results

**Fig. 7:**
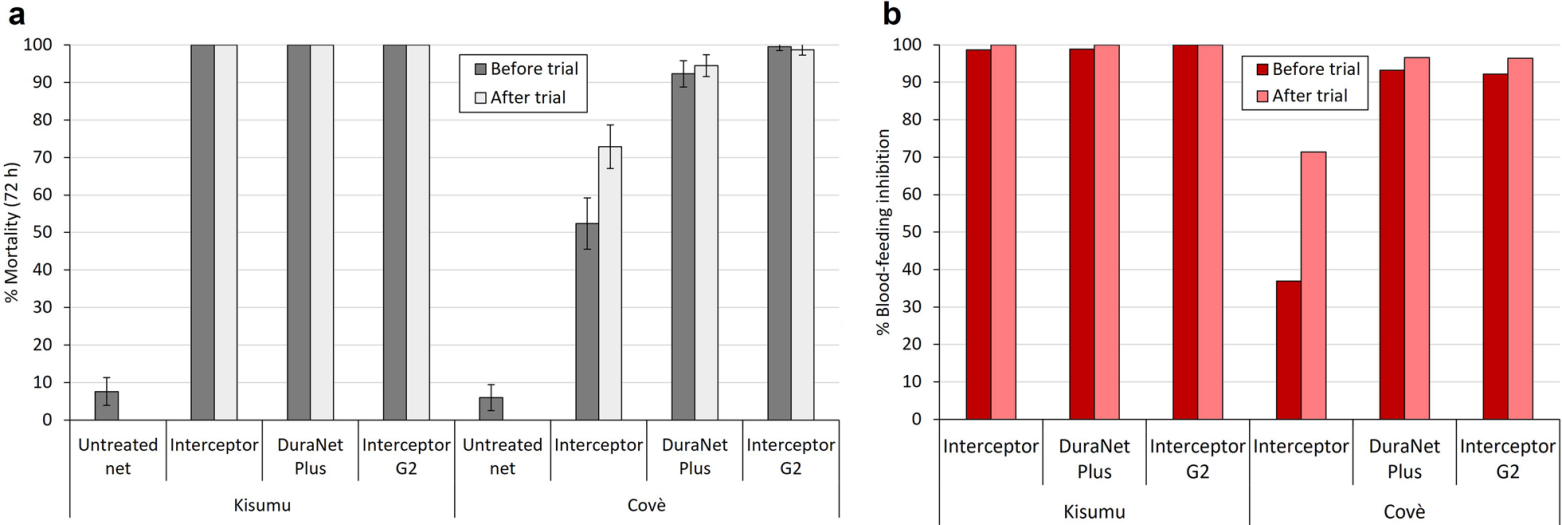
Mortality (**a**) and blood-feeding inhibition (**b**) of susceptible *Anopheles gambiae s.s.* Kisumu strain and pyrethroid-resistant *An. gambiae s.l.* Covè strain exposed to net pieces cut from whole nets before and after experimental hut trial 1 in supplementary tunnel tests. Approximately 100 mosquitoes were exposed to each of two randomly selected net pieces from each treatment arm in one replicate tunnel test. Error bars represent 95% confidence intervals

**Fig. 8:**
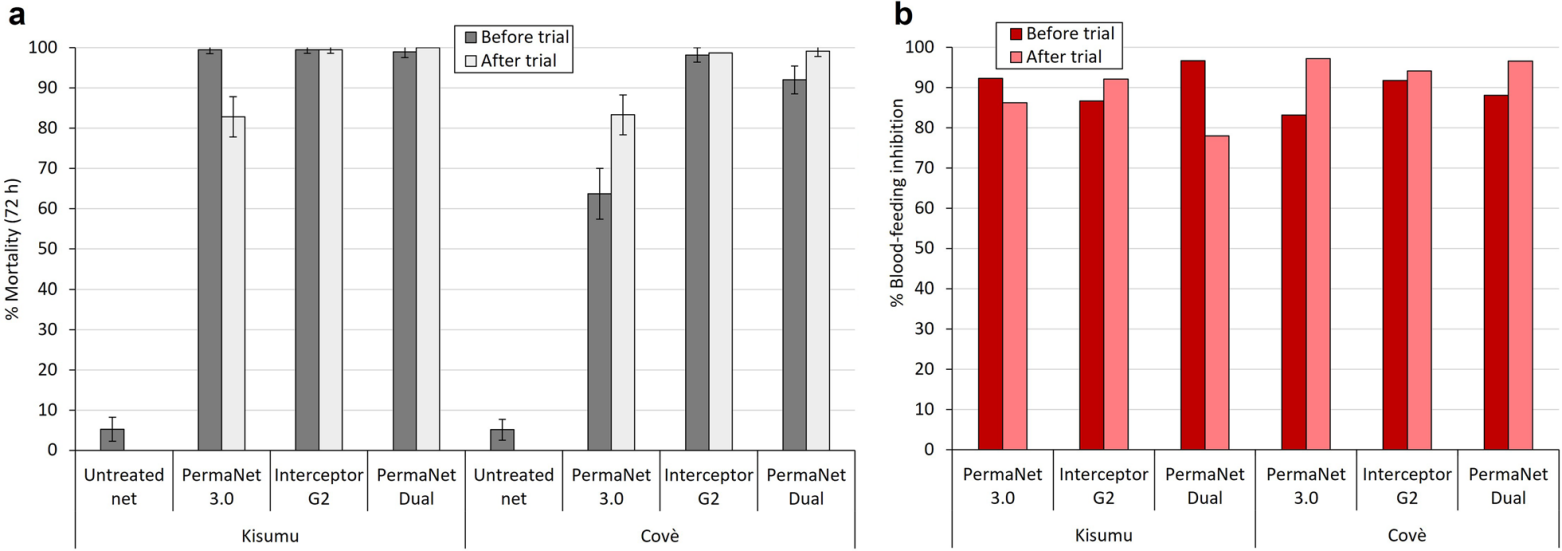
Mortality (**a**) and blood-feeding inhibition (**b**) of susceptible *Anopheles gambiae s.s.* Kisumu strain and pyrethroid-resistant *An. gambiae s.l.* Covè strain exposed to net pieces cut from whole nets before and after experimental hut trial 2 in supplementary tunnel tests. Approximately 100 mosquitoes were exposed to each of two randomly selected net pieces from each treatment arm in one replicate tunnel test. Error bars represent 95% confidence intervals

Net pieces taken from ITNs before and after both experimental hut trials induced high mortality (> 80%) and blood-feeding inhibition (> 75%) of the susceptible Kisumu strain (Figs. 7, 8). Against the pyrethroid-resistant Covè strain, mortality and blood-feeding inhibition were lowest with the pyrethroid-only ITN (Interceptor^®^), failing to surpass 75% for both outcomes. The pyrethroid-PBO ITNs (DuraNet^®^ Plus and PermaNet^®^ 3.0) induced comparatively higher mortality (64–95%) and blood-feeding inhibition (81–97%). The highest levels of mortality (> 98%) and blood-feeding inhibition (> 90%) were recorded with the pyrethroid-chlorfenapyr ITNs (Interceptor^®^ G2 and PermaNet^®^ Dual), thus corroborating the experimental hut results demonstrating the superior efficacy of this ITN class against pyrethroid-resistant mosquitoes. Results for each ITN type and strain were generally similar between net pieces taken before and after the hut trials. Detailed tunnel test results are provided as supplementary information (Additional file 1: Tables S5, S6).

## Discussion

PBO inhibits mosquito P450 enzymes associated with pyrethroid resistance while CFP is a pro-insecticide requiring activation by P450s. It has thus been hypothesized that pyrethroid-PBO ITNs may reduce the performance of pyrethroid-CFP ITNs when these ITN types are combined in the same household. This is a realistic scenario in areas where NMCPs have distributed pyrethroid-CFP and pyrethroid-PBO ITNs in the same areas through separate distribution channels. To investigate this, experimental hut trials were performed to evaluate the impact of pyrethroid-CFP ITNs both when applied alone and when combined with pyrethroid-PBO ITNs against a pyrethroid-resistant vector population in southern Benin. The results show evidence that the performance of pyrethroid-CFP ITNs was reduced when they were combined with pyrethroid-PBO ITNs compared to when they were applied alone. These findings suggest that in similar settings, prioritizing deployment of pyrethroid-CFP ITNs would maximize vector control impact.

The low mortality observed with the pyrethroid-only ITN (Interceptor^®^) in this study is consistent with previous studies conducted against this vector population [26, 30, 31] and is attributable to the high intensity of pyrethroid resistance as confirmed by the susceptibility bioassays in this study. Although pre-exposure to PBO partially restored pyrethroid susceptibility in these bioassays, the improvements in vector mortality observed with the pyrethroid-PBO ITNs relative to the pyrethroid-only ITN were modest. This corroborates results from several experimental hut trials in West Africa demonstrating poor or no improvement in mosquito mortality with pyrethroid-PBO ITNs relative to pyrethroid-only ITNs [30, 32, 33], and reiterates the need for cRCTs to establish the public health value of pyrethroid-PBO ITNs in the region. In contrast, markedly improved mortality rates were observed with both types of pyrethroid-CFP ITN compared to pyrethroid-PBO and pyrethroid-only ITNs, which can be attributed to the susceptibility of the Covè vector population to CFP. The superiority of pyrethroid-CFP ITNs over other ITN types is supported by recent experimental hut trials [29, 34–36] and cRCTs in Benin [13] and Tanzania [14], and was formally acknowledged in recently revised WHO guidelines recommending them over pyrethroid-only and pyrethroid-PBO ITNs [10].

In both experimental hut trials, vector mortality was improved with combinations that involved pyrethroid-CFP ITNs compared to those that did not and the highest levels of mortality were achieved with combinations of two pyrethroid-CFP ITNs. This finding together with the high mortality rates observed with pyrethroid-CFP ITNs applied alone suggests that optimal malaria transmission control would be achieved when households and communities receive pyrethroid-CFP ITNs alone rather than in combination with other ITN types. Given that pyrethroid-CFP ITNs induced higher mortality than pyrethroid-PBO ITNs when applied alone, the reduced performance of the pyrethroid-CFP ITN plus pyrethroid-PBO ITN combinations compared to two pyrethroid-CFP ITNs may simply be due to the reduction in the overall surface area of pyrethroid-CFP ITN. However, the fact that mortality was lower with the pyrethroid-CFP ITN plus pyrethroid-PBO ITN combinations compared to the pyrethroid-CFP ITNs applied singly suggests there may have been a negative interaction in the combination which reduced the killing effect of the pyrethroid-CFP ITNs.

The reduced performance of pyrethroid-CFP ITNs when combined with pyrethroid-PBO ITNs could be attributable to antagonism between CFP and PBO as observed in the interaction bioassays performed with mosquitoes collected from the Covè hut site and in previous laboratory studies [18–22]. However, higher exiting was also observed in huts combining pyrethroid-CFP ITNs with pyrethroid-PBO ITNs relative to huts with two pyrethroid-CFP ITNs. This was probably due to the higher dose and surface availability of pyrethroids in the pyrethroid-PBO ITNs. This increased early exiting of mosquitoes elicited by the excitorepellency of the pyrethroid could have also reduced mosquito contact with pyrethroid-CFP ITNs when combined with the pyrethroid-PBO ITNs thus compromising their impact. Despite this, the pyrethroid-CFP ITN performed better in combination with the pyrethroid-PBO ITN relative to the pyrethroid-only ITN in the first hut trial, which could be attributed to the higher efficacy of the pyrethroid-PBO ITN component against pyrethroid-resistant mosquitoes that did not contact both ITNs.

The findings from this study provide useful insights for NMCPs considering co-deployment of pyrethroid-CFP and pyrethroid-PBO ITNs through mass campaigns or multiple distribution channels. To help guide optimal ITN deployment strategy, separate scenarios can be considered where high coverage of one ITN type has been achieved via mass campaigns and the impact of introducing another ITN type via continuous distribution channels such as through schools and antenatal clinics. In the first scenario, where high coverage of pyrethroid-CFP ITNs has already been achieved, the results of this study suggest that introducing pyrethroid-PBO or pyrethroid-only ITNs through continuous channels would result in lower levels of vector mortality and, therefore, transmission control relative to deployment of pyrethroid-CFP ITNs alone. In the opposite scenario however, where pyrethroid-PBO or pyrethroid-only ITNs are already present at high coverage, introducing pyrethroid-CFP ITNs would cause marked improvements in vector mortality and transmission control. Thus, in all scenarios, the findings suggest that malaria transmission control impact could be maximized by prioritizing distribution of pyrethroid-CFP ITNs. Blanket coverage of pyrethroid-CFP ITNs may, however, accelerate the evolution of CFP resistance. NMCPs should therefore consider appropriate insecticide resistance management strategies, such as co-deployment of pyrethroid-CFP ITNs with unrelated indoor residual spraying (IRS) insecticides or rotational distribution of ITN classes delivering different insecticides, to delay resistance and maximize the lifespan of CFP for malaria vector control.

Although the difference in mosquito mortality observed between the hut treatments were statistically significant, the absolute differences were however moderate. Hence, it is unclear whether the findings would be operationally significant when implemented at scale. Transmission dynamics models, used to predict the epidemiological impact of ITNs and IRS based on experimental hut data [24, 37–39], may help provide greater insights into the malaria control potential of different ITN co-deployment strategies. Controlled laboratory assays investigating metabolic and behavioural responses of mosquitoes exposed to pyrethroid-CFP ITNs alone and in combination with pyrethroid-PBO ITNs could also help elucidate the role of P450 inhibition in mediating the interactions observed in experimental huts.

Other next-generation ITN and IRS products being developed for malaria control contain active ingredients which are activated or detoxified by P450s and thus have potential to interact positively and negatively with pyrethroid-PBO ITNs when deployed together. Notably, some insecticides developed for use as IRS products, such as pirimiphos-methyl, are bioactivated by P450s and could therefore interact negatively with PBO [40, 41]. A recent study conducted at the same experimental hut site in Benin lends credence to this hypothesis, showing reduced efficacy of pirimiphos-methyl IRS when combined with pyrethroid-PBO ITNs [42]. As coverage of next-generation vector control increases, more studies like this one, investigating the impact of combining novel ITN and IRS products, are needed to inform optimal co-deployment policy.

## Conclusions

This study shows reduced performance of pyrethroid-CFP ITNs when combined with pyrethroid-PBO ITNs compared to when applied alone. Evidence from interaction bioassays suggest this was partly attributable to antagonism between CFP and PBO however, enhanced excitorepellency of the synergized pyrethroid in the pyrethroid-PBO ITNs could have also contributed. Higher levels of vector mortality were observed with combinations that involved pyrethroid-CFP ITNs compared to those that did not and the highest mortality was achieved when pyrethroid-CFP ITNs were applied singly or as two nets together. These findings suggest that in similar contexts, prioritizing distribution of pyrethroid-CFP ITNs over other ITN types would maximize vector control impact.

## Supplementary Information

**Additional file 1: Figure S1.** Design of West African experimental huts. Credit: Hougard et al. 2007. **Figure S2.** Division of experimental huts into two equally sized sleeping areas for net combination treatment arms. **Table S1.** Chemical content of net pieces cut from whole insecticide-treated nets before and after the experimental hut trials. *World Health Organization tolerance threshold is ±25%. **Table S2.** Resistance bioassay results with F1 progeny of *Anopheles gambiae* sensu lato collected from the experimental hut site in Covè during trial 1. Approximately 100 mosquitoes aged 3–5 days were exposed to each treatment arm for 60 mins in four replicates of 20–25. **Table S3.** Resistance bioassay results with F1 progeny of *Anopheles gambiae* sensu lato collected from the experimental hut site in Covè during trial 2. Approximately 100 mosquitoes aged 3–5 days were exposed to each treatment arm for 60 mins in four replicates of 20–25. **Table S4.** Chlorfenapyr and piperonyl butoxide (PBO) interaction bioassay results with the pyrethroid-resistant *Anopheles gambiae* sensu lato Covè strain. Approximately 150 mosquitoes aged 3–5 days were exposed to each dose of chlorfenapyr for 60 mins with and without pre-exposure to the discriminating dose of PBO in 6 replicates of 20–25. **Table S5.** Supplementary tunnel test results with susceptible *Anopheles gambiae* sensu stricto Kisumu strain and pyrethroid-resistant *Anopheles gambiae* sensu lato Covè strain exposed to net pieces cut from whole nets before and after experimental hut trial 1. Approximately 200 mosquitoes aged 5–8 days were exposed to each treatment arm in two replicate tunnel tests. **Table S6.** Supplementary tunnel test results with susceptible *Anopheles gambiae* sensu stricto Kisumu strain and pyrethroid-resistant *Anopheles gambiae* sensu lato Covè strain exposed to net pieces cut from whole nets before and after experimental hut trial 2. Approximately 200 mosquitoes aged 5–8 days were exposed to each treatment arm in two replicate tunnel tests.

## Data Availability

The datasets used and/or analysed during the current study are available from the corresponding author on reasonable request.

## Abbreviations

CFP: Chlorfenapyr
cRCT: Cluster-randomized controlled trial
CREC: Centre de Recherche Entomologique de Cotonou
DC: Discriminating concentration
ITN: Insecticide-treated net
LSHTM: London School of Hygiene & Tropical Medicine
NMCP: National malaria control programme
PBO: Piperonyl butoxide
P450: Cytochrome P450 monooxygenase
WHO: World Health Organization
