# Supplementary material for: Can the performance of pyrethroid-chlorfenapyr nets be reduced when combined with pyrethroid-piperonyl butoxide (PBO) nets?
Source: Malar J. 2023 Jul 21;22:214. doi: 10.1186/s12936-023-04648-6 (PMC10362717; doi:10.1186/s12936-023-04648-6)
Supplement: Supplementary file 1 — Additional file 1: Figure S1. Design of West African experimental huts. Credit: Hougard et al. 2007. Figure S2. Division of experimental huts into two equally sized sleeping areas for net combination treatment arms. Table S1. Chemical content of net pieces cut from whole insecticide-treated nets before and after the experimental hut trials. *World Health Organization tolerance threshold is ±25%. Table S2. Resistance bioassay results with F1 progeny of Anopheles gambiae sensu lato collected from the experimental hut site in Covè during trial 1. Approximately 100 mosquitoes aged 3–5 days were exposed to each treatment arm for 60 mins in four replicates of 20–25. Table S3. Resistance bioassay results with F1 progeny of Anopheles gambiae sensu lato collected from the experimental hut site in Covè during trial 2. Approximately 100 mosquitoes aged 3–5 days were exposed to each treatment arm for 60 mins in four replicates of 20–25. Table S4. Chlorfenapyr and piperonyl butoxide (PBO) interaction bioassay results with the pyrethroid-resistant Anopheles gambiae sensu lato Covè strain. Approximately 150 mosquitoes aged 3–5 days were exposed to each dose of chlorfenapyr for 60 mins with and without pre-exposure to the discriminating dose of PBO in 6 replicates of 20–25. Table S5. Supplementary tunnel test results with susceptible Anopheles gambiae sensu stricto Kisumu strain and pyrethroid-resistant Anopheles gambiae sensu lato Covè strain exposed to net pieces cut from whole nets before and after experimental hut trial 1. Approximately 200 mosquitoes aged 5–8 days were exposed to each treatment arm in two replicate tunnel tests. Table S6. Supplementary tunnel test results with susceptible Anopheles gambiae sensu stricto Kisumu strain and pyrethroid-resistant Anopheles gambiae sensu lato Covè strain exposed to net pieces cut from whole nets before and after experimental hut trial 2. Approximately 200 mosquitoes aged 5–8 days were exposed to each treatment arm in two replicate t [file 12936_2023_4648_MOESM1_ESM.docx]

**Additionl file**

**Can the performance of pyrethroid-chlorfenapyr nets be reduced when combined with pyrethroid-piperonyl butoxide (PBO) nets?**

Thomas Syme^1,2,3*^, Judicaël Nounagnon^2,3^, Boris N’dombidjé^2,3^, Martial Gbegbo^2,3^, Abel Agbevo^2,3^, Juniace Ahoga^2,3^, Corine Ngufor^1,2,3*^

^1^London School of Hygiene & Tropical Medicine, United Kingdom

^2^Centre de Recherche Entomologique de Cotonou, Benin

^3^Pan African Malaria Vector Research Consortium (PAMVERC), Benin

*Corresponding authors

(CN) Email: [corine.ngufor@lshtm.ac.uk](mailto:corine.ngufor@lshtm.ac.uk)

(TS) Email: [Thomas.syme@lshtm.ac.uk](mailto:Thomas.syme@lshtm.ac.uk)

**Key words:** *Insecticide-treated nets, chlorfenapyr, piperonyl butoxide, pyrethroid, vector control, Anopheles gambiae, mosquitoes, malaria, experimental huts, pro-insecticide, antagonism*


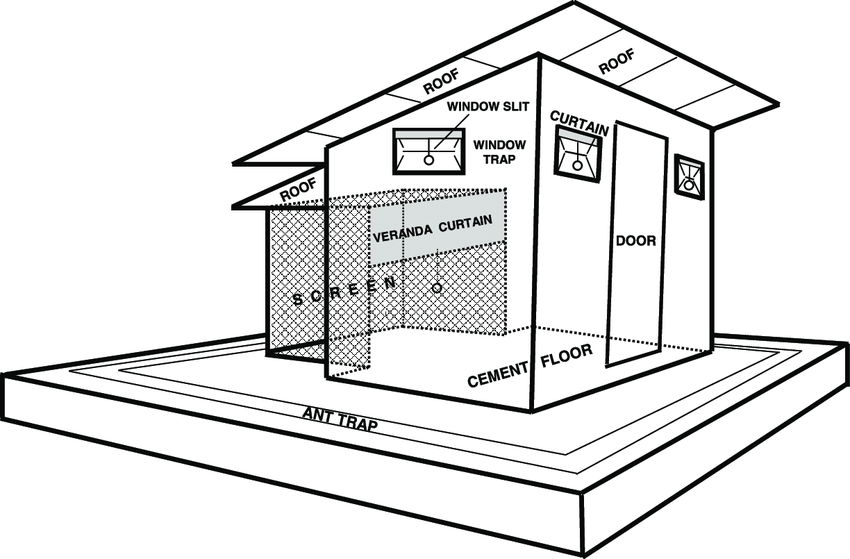


**Figure S1:** Design of West African experimental huts. Credit: Hougard et al. 2007.


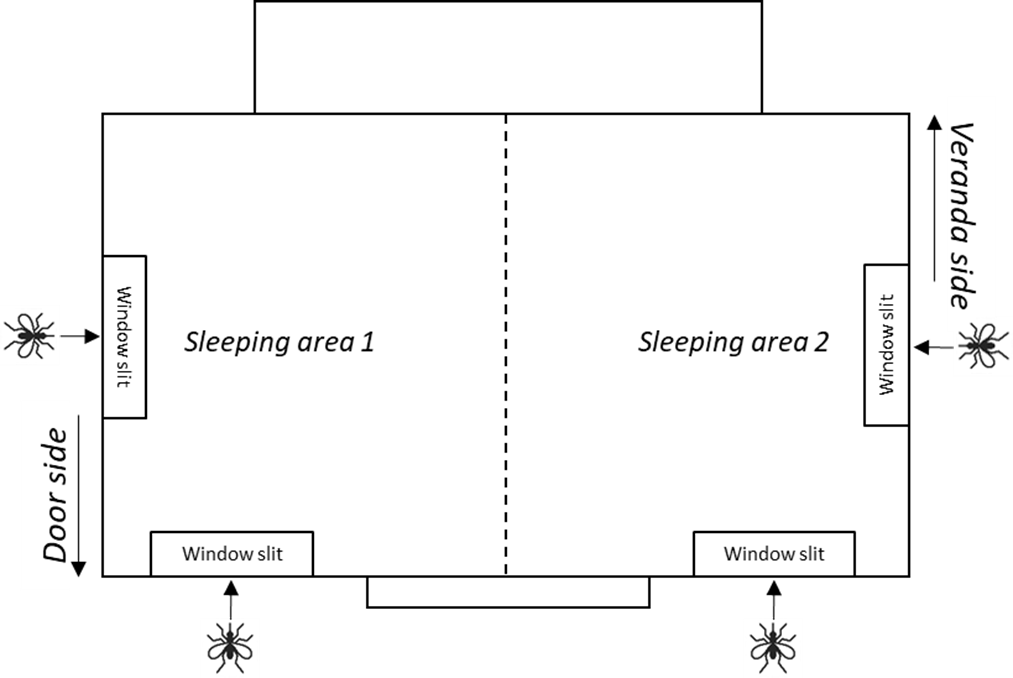


**Figure S2:** Division of experimental huts into two equally sized sleeping areas for net combination treatment arms.

**Table S1:** Chemical content of net pieces cut from whole insecticide-treated nets before and after the experimental hut trials. **World Health Organisation tolerance threshold is ±25%*

| **Trial** | **ITN type** | **Active ingredients/**  **Synergists** | **Target chemical content (g/kg)** | **Chemical content (g/kg)** | | | | **Within net variation (%RSD)** | |
| --- | --- | --- | --- | --- | --- | --- | --- | --- | --- |
|  |  |  |  | **Before trial** | **% Difference (±)** | **After trial** | **% Difference (±)** | **Before trial** | **After trial** |
| Trial 1 | Interceptor | Alpha-cypermethrin | 5.0 | 5.3 | +6.0 | 5.9 | +18.0 | 15.5 | 11.1 |
|  | DuraNet Plus | Alpha-cypermethrin | 6.0 | 5.9 | ­­̶ 1.7 | 5.7 | ̶ 5.0 | 2.8 | 2.8 |
|  |  | Piperonyl butoxide | 2.2 | 3.0 | +36.4 | 2.6 | +18.2 | 3.8 | 3.3 |
|  | Interceptor G2 | Alpha-cypermethrin | 2.4 | 2.6 | +8.3 | 2.6 | +8.3 | 21.0 | 19.5 |
|  |  | Chlorfenapyr | 4.8 | 4.6 | ̶ 4.2 | 4.3 | ̶ 10.4 | 22.3 | 22.3 |
| Trial 2 | PermaNet 3.0 (sides) | Deltamethrin | 2.1 | 2.0 | -4.8 | 2.0 | -4.8 | 3.4 | 4.7 |
|  | PermaNet 3.0 (roof) | Deltamethrin | 4.0 | 3.8 | -5.0 | 3.8 | -5.0 | 1.2 | 1.2 |
|  |  | Piperonyl butoxide | 25.0 | 26.3 | +5.2 | 19.0 | -24.0 | 0.4 | 3.8 |
|  | Interceptor G2 | Alpha-cypermethrin | 2.4 | 2.9 | +20.8 | 2.9 | +20.8 | 17.1 | 12.7 |
|  |  | Chlorfenapyr | 4.8 | 5.1 | +6.3 | 4.9 | +2.1 | 15.0 | 19.7 |
|  | PermaNet Dual | Deltamethrin | 2.1 | 1.9 | -9.5 | 2.4 | +14.3 | 4.1 | 9.9 |
|  |  | Chlorfenapyr | 5.0 | 5.6 | +12.0 | 4.5 | -10.0 | 4.1 | 7.2 |

**Table S2:** Resistance bioassay results with F1 progeny of *Anopheles gambiae sensu lato* collected from the experimental hut site in Covè during trial 1. *Approximately 100 mosquitoes aged 3–5 days were exposed to each treatment arm for 60 mins in four replicates of 20–25.*

| **Treatment** | **Acetone (control)** | **PBO** | **Alpha-cypermethrin** | | | | **PBO + Alpha-cypermethrin** | **Chlorfenapyr** |
| --- | --- | --- | --- | --- | --- | --- | --- | --- |
| **Dose** | **̶** | **400 µg** | **12.5 µg (1x)** | **25 µg (2x)** | **62.5 µg (5x)** | **125 µg (10x)** | **400 µg + 12.5 µg** | **100 µg (1x)** |
| **N** | 93 | 92 | 97 | 99 | 99 | 97 | 89 | 100 |
| **N KD 60 mins** | 0 | 0 | 89 | 82 | 85 | 78 | 83 | 31 |
| **% KD 60 mins** | 0.0 | 0.0 | 91.8 | 82.8 | 85.9 | 80.4 | 93.3 | 31.0 |
| **95% CIs** | ̶ | ̶ | 86.2–97.4 | 75.1–90.5 | 78.8–93.0 | 72.3–88.5 | 88.2–98.4 | 21.6–40.4 |
| **N dead 24 h** | 0 | 0 | 16 | 66 | 70 | 70 | 89 | 95 |
| **% dead 24 h** | 0.0 | 0.0 | 16.5 | 66.7 | 70.7 | 72.2 | 100 | 95.0 |
| **95% CIs** | ̶ | ̶ | 9.1–23.9 | 57.4–76.0 | 61.7–79.7 | 63.3–81.1 | ̶ | 90.7–99.3 |
| **% dead 24 h (corrected)** | ̶ | 0.0 | 16.5 | 66.7 | 70.7 | 72.2 | 100 | 95.0 |
| **N dead 48 h** | 2 | ̶ | ̶ | ̶ | ̶ | ̶ | ̶ | 97 |
| **% dead 48 h** | 2.2 | ̶ | ̶ | ̶ | ̶ | ̶ | ̶ | 97.0 |
| **95% CIs** | 0–5.2 | ̶ | ̶ | ̶ | ̶ | ̶ | ̶ | 93.7–100 |
| **% dead 48 h (corrected)** | ̶ | ̶ | ̶ | ̶ | ̶ | ̶ | ̶ | 96.9 |
| **N dead 72 h** | 2 | ̶ | ̶ | ̶ | ̶ | ̶ | ̶ | 97 |
| **% dead 72 h** | 2.2 | ̶ | ̶ | ̶ | ̶ | ̶ | ̶ | 97.0 |
| **95% CIs** | 0–5.2 | ̶ | ̶ | ̶ | ̶ | ̶ | ̶ | 93.7–100 |
| **% dead 72 h (corrected)** | ̶ | ̶ | ̶ | ̶ | ̶ | ̶ | ̶ | 96.9 |

**Table S3:** Resistance bioassay results with F1 progeny of *Anopheles gambiae sensu lato* collected from the experimental hut site in Covè during trial 2. *Approximately 100 mosquitoes aged 3–5 days were exposed to each treatment arm for 60 mins in four replicates of 20–25.*

| **Test method** | **Tube tests** | | | | | | **Bottle bioassays** | |
| --- | --- | --- | --- | --- | --- | --- | --- | --- |
| **Treatment** | **Silicone oil (control)** | **PBO** | **Deltamethrin** | | | **PBO + Deltamethrin** | **Acetone (control)** | **Chlorfenapyr** |
| **Dose** | **̶** | **4%** | **0.05% (1x)** | **0.25% (5x)** | **0.5% (10x)** | **4% + 0.05%** | **̶** | **100 µg (1x)** |
| **N** | 97 | 98 | 96 | 85 | 100 | 93 | 100 | 101 |
| **N KD 60 mins** | 1 | 0 | 2 | 73 | 100 | 44 | 0 | 21 |
| **% KD 60 mins** | 1.0 | 0.0 | 2.1 | 85.9 | 100 | 47.3 | 0.0 | 20.8 |
| **95% CIs** | 0–3.0 | ̶ | 0–5.0 | 78.8–93.0 | ̶ | 37.2–57.4 | ̶ | 12.6–29.0 |
| **N dead 24 h** | 3 | 0 | 10 | 45 | 86 | 55 | 0 | 96 |
| **% dead 24 h** | 3.1 | 0.0 | 10.4 | 52.9 | 86.0 | 59.1 | 0.0 | 95.0 |
| **95% CIs** | -0.3-6.5 | ̶ | 4.3–16.5 | 42.3–63.5 | 79.2–92.8 | 49.1–69.1 | ̶ | 90.7–99.3 |
| **% dead 24 h (corrected)** | ̶ | 0.0 | 7.5 | 51.4 | 85.6 | 57.8 | ̶ | 95.0 |
| **N dead 48 h** | ̶ | ̶ | ̶ | ̶ | ̶ | ̶ | 0 | 99 |
| **% dead 48 h** | ̶ | ̶ | ̶ | ̶ | ̶ | ̶ | 0.0 | 98.0 |
| **95% CIs** | ̶ | ̶ | ̶ | ̶ | ̶ | ̶ | ̶ | 95.3–100 |
| **% dead 48 h (corrected)** | ̶ | ̶ | ̶ | ̶ | ̶ | ̶ | ̶ | 98.0 |
| **N dead 72 h** | ̶ | ̶ | ̶ | ̶ | ̶ | ̶ | 0 | 101 |
| **% dead 72 h** | ̶ | ̶ | ̶ | ̶ | ̶ | ̶ | 0.0 | 100 |
| **95% CIs** | ̶ | ̶ | ̶ | ̶ | ̶ | ̶ | ̶ | ̶ |
| **% dead 72 h (corrected)** | ̶ | ̶ | ̶ | ̶ | ̶ | ̶ | ̶ | 100 |

**Table S4:** Chlorfenapyr and piperonyl butoxide (PBO) interaction bioassay results with the pyrethroid-resistant *Anopheles gambiae sensu lato* Covè strain. *Approximately 150 mosquitoes aged 3–5 days were exposed to each dose of chlorfenapyr for 60 mins with and without pre-exposure to the discriminating dose of PBO in 6 replicates of 20–25.*

| **Insecticide** | **Acetone (control)** | **PBO** | **Chlorfenapyr** | | | | **PBO + Chlorfenapyr** | | | |
| --- | --- | --- | --- | --- | --- | --- | --- | --- | --- | --- |
| **Dose** | **̶** | **400 µg** | **25 µg** | **50 µg** | **75 µg** | **100 µg** | **400 µg + 25 µg** | **400 µg + 50 µg** | **400 µg + 75 µg** | **400 µg + 100 µg** |
| **N** | 289 | 242 | 141 | 137 | 139 | 134 | 122 | 135 | 129 | 125 |
| **N KD 60 mins** | 0 | 0 | 0 | 0 | 0 | 0 | 0 | 0 | 0 | 0 |
| **% KD 60 mins** | 0 | 0 | 0 | 0 | 0 | 0 | 0 | 0 | 0 | 0 |
| **95% CIs** | ̶ | ̶ | ̶ | ̶ | ̶ | ̶ | ̶ | ̶ | ̶ | ̶ |
| **N dead 24 h** | 3 | 4 | 113 | 108 | 81 | 103 | 13 | 53 | 50 | 48 |
| **% dead 24 h** | 1.0 | 1.7 | 80.1 | 78.8 | 58.3 | 76.9 | 10.7 | 39.3 | 38.8 | 38.4 |
| **95% CIs** | 0–2.2 | 0–3.3 | 73.6–86.7 | 72.0–85.7 | 50.1–66.5 | 69.7–84.0 | 5.2–16.1 | 31.0–47.5 | 30.4–47.0 | 29.9–46.9 |
| **% dead 24 h (corrected)** | ̶ | 0.6 | 79.9 | 78.6 | 57.8 | 76.6 | 9.7 | 38.6 | 38.1 | 37.8 |
| **N dead 48 h** | 6 | 5 | 115 | 116 | 100 | 121 | 24 | 71 | 80 | 69 |
| **% dead 48 h** | 2.1 | 2.1 | 81.6 | 84.7 | 71.9 | 90.3 | 19.7 | 52.6 | 62.0 | 55.2 |
| **95% CIs** | 0.4–3.7 | 0.3–3.9 | 75.2–88.0 | 78.6–90.7 | 64.5–79.4 | 85.3–95.3 | 12.6–26.7 | 44.2–61.0 | 53.6–70.4 | 46.5–63.9 |
| **% dead 48 h (corrected)** | ̶ | 0 | 81.2 | 84.3 | 71.3 | 90.1 | 18.0 | 51.6 | 61.2 | 54.3 |
| **N dead 72 h** | 6 | 6 | 123 | 125 | 115 | 126 | 52 | 93 | 99 | 100 |
| **% dead 72 h** | 2.1 | 2.5 | 87.2 | 91.2 | 82.7 | 94.0 | 42.6 | 68.9 | 76.7 | 80.0 |
| **95% CIs** | 0.4–3.7 | 0.5–4.4 | 81.7–92.7 | 86.5–96.0 | 76.5–89.0 | 90.0–98.0 | 33.9–51.4 | 61.1–76.7 | 69.5–84.0 | 73.0–87.0 |
| **% dead 72 h (corrected)** | ̶ | 0.4 | 87.0 | 91.1 | 82.4 | 93.9 | 41.4 | 68.2 | 76.3 | 79.6 |

**Table S5:** Supplementary tunnel test results with susceptible *Anopheles gambiae sensu stricto* Kisumu strain and pyrethroid-resistant *Anopheles gambiae sensu lato* Covè strain exposed to net pieces cut from whole nets before and after experimental hut trial 1. *Approximately 200 mosquitoes aged 5–8 days were exposed to each treatment arm in two replicate tunnel tests.*

| **Strain** | **Kisumu** | | | | | | | **Covè** | | | | | | |
| --- | --- | --- | --- | --- | --- | --- | --- | --- | --- | --- | --- | --- | --- | --- |
| **Net type** | **Untreated net** | **Interceptor** | | **DuraNet Plus** | | **Interceptor G2** | | **Untreated net** | **Interceptor** | | **DuraNet Plus** | | **Interceptor G2** | |
| **Net status** | **̶** | **Before trial** | **After trial** | **Before trial** | **After trial** | **Before trial** | **After trial** | **̶** | **Before trial** | **After trial** | **Before trial** | **After trial** | **Before trial** | **After trial** |
| **N exposed** | 198 | 185 | 184 | 207 | 180 | 229 | 169 | 182 | 206 | 225 | 221 | 238 | 194 | 235 |
| **N pass** | 139 | 47 | 60 | 50 | 57 | 94 | 57 | 98 | 105 | 137 | 62 | 115 | 31 | 96 |
| **% pass** | 70.2 | 25.4 | 32.6 | 24.2 | 31.7 | 41.0 | 33.7 | 53.8 | 51.0 | 60.9 | 28.1 | 48.3 | 16.0 | 40.9 |
| **95% CIs** | 63.8-76.6 | 19.1-31.7 | 25.8-39.4 | 18.4-30.0 | 24.9-38.5 | 34.6-47.4 | 26.6-40.8 | 46.6-61.0 | 44.2-57.8 | 54.5-67.3 | 22.2-34.0 | 42.0-54.6 | 10.8-21.2 | 34.6-47.2 |
| **N blf** | 174 | 2 | 0 | 2 | 0 | 0 | 0 | 133 | 95 | 47 | 11 | 6 | 11 | 6 |
| **% blf** | 87.9 | 1.1 | 0 | 1.0 | 0 | 0 | 0 | 73.1 | 46.1 | 20.9 | 5.0 | 2.5 | 5.7 | 2.6 |
| **95% CIs** | 83.4-92.4 | 0-2.6 | ̶ | 0-2.4 | ̶ | ̶ | ̶ | 66.7-79.5 | 39.3-52.9 | 15.6-26.2 | 2.1-7.9 | 0.5-4.5 | 2.4-9.0 | 0.6-4.6 |
| **% blf inhibition** | - | 98.7 | 100 | 98.9 | 100 | 100 | 100 | 16.8 | 47.6 | 76.2 | 94.3 | 97.2 | 93.5 | 97.0 |
| **N dead imm** | 12 | 182 | 184 | 207 | 180 | 229 | 169 | 8 | 78 | 123 | 197 | 213 | 186 | 198 |
| **% dead imm** | 6.1 | 98.4 | 100 | 100 | 100 | 100 | 100 | 4.4 | 37.9 | 54.7 | 89.1 | 89.5 | 95.9 | 84.3 |
| **95% CIs** | 2.8-9.4 | 96.6-100 | ̶ | ̶ | ̶ | ̶ | ̶ | 1.4-7.4 | 31.3-44.5 | 48.2-61.2 | 85.0-93.2 | 85.6-93.4 | 93.1-98.7 | 79.6-89.0 |
| **% corrected dead imm** | ̶ | 98.3 | 100 | 100 | 100 | 100 | 100 | ̶ | 35.0 | 52.6 | 88.6 | 89.0 | 95.7 | 83.6 |
| **N dead 24 h** | 14 | 185 | 184 | 207 | 180 | 229 | 169 | 8 | 97 | 153 | 203 | 223 | 193 | 229 |
| **% dead 24 h** | 7.1 | 100 | 100 | 100 | 100 | 100 | 100 | 4.4 | 47.1 | 68.0 | 91.9 | 93.7 | 99.5 | 97.4 |
| **95% CIs** | 3.5-10.7 | ̶ | ̶ | ̶ | ̶ | ̶ | ̶ | 1.4-7.4 | 40.3-53.9 | 61.9-74.1 | 88.3-95.5 | 90.6-96.8 | 98.5-100 | 95.4-99.4 |
| **% corrected dead 24 h** | ̶ | 100 | 100 | 100 | 100 | 100 | 100 | ̶ | 44.7 | 66.5 | 91.5 | 93.4 | 99.5 | 97.3 |
| **N dead 48 h** | 15 | 185 | 184 | 207 | 180 | 229 | 169 | 9 | 106 | 158 | 204 | 224 | 193 | 230 |
| **% dead 48h** | 7.6 | 100 | 100 | 100 | 100 | 100 | 100 | 4.9 | 51.5 | 70.2 | 92.3 | 94.1 | 99.5 | 97.9 |
| **95% CIs** | 3.9-11.3 | ̶ | ̶ | ̶ | ̶ | ̶ | ̶ | 1.8-8.0 | 44.7-58.3 | 64.2-76.2 | 88.8-95.8 | 91.1-97.1 | 98.5-100.5 | 96.1-99.7 |
| **% corrected dead 48 h** | ̶ | 100 | 100 | 100 | 100 | 100 | 100 | ̶ | 49.0 | 68.7 | 91.9 | 93.8 | 99.5 | 97.8 |
| **N dead 72 h** | 15 | 185 | 184 | 207 | 180 | 229 | 169 | 11 | 108 | 164 | 204 | 225 | 193 | 232 |
| **% dead 72 h** | 7.6 | 100 | 100 | 100 | 100 | 100 | 100 | 6.0 | 52.4 | 72.9 | 92.3 | 94.5 | 99.5 | 98.7 |
| **95% CIs** | 3.9-11.3 | ̶ | ̶ | ̶ | ̶ | ̶ | ̶ | 2.5-9.5 | 45.6-59.2 | 67.1-78.7 | 88.8-95.8 | 91.6-97.4 | 98.5-100 | 97.3-100 |
| **% corrected dead 72 h** | ̶ | 100 | 100 | 100 | 100 | 100 | 100 | ̶ | 49.4 | 71.2 | 91.8 | 94.1 | 99.5 | 98.6 |

**Table S6:** Supplementary tunnel test results with susceptible *Anopheles gambiae sensu stricto* Kisumu strain and pyrethroid-resistant *Anopheles gambiae sensu lato* Covè strain exposed to net pieces cut from whole nets before and after experimental hut trial 2. *Approximately 200 mosquitoes aged 5–8 days were exposed to each treatment arm in two replicate tunnel tests.*

| **Strain** | **Kisumu** | | | | | | | **Covè** | | | | | | | |
| --- | --- | --- | --- | --- | --- | --- | --- | --- | --- | --- | --- | --- | --- | --- | --- |
| **Net type** | **Untreated net** | **PermaNet 3.0** | | **Interceptor G2** | | **PermaNet Dual** | | **Untreated net** | **PermaNet 3.0** | | **Interceptor G2** | | **PermaNet Dual** | |  |
| **Net status** | **̶** | **Before trial** | **After trial** | **Before trial** | **After trial** | **Before trial** | **After trial** | **̶** | **Before trial** | **After trial** | **Before trial** | **After trial** | **Before trial** | **After trial** |  |
| **N exposed** | 211 | 191 | 221 | 220 | 213 | 206 | 221 | 275 | 223 | 215 | 221 | 223 | 225 | 218 |  |
| **N pass** | 188 | 59 | 106 | 21 | 79 | 33 | 138 | 242 | 59 | 87 | 62 | 64 | 71 | 54 |  |
| **% pass** | 89.1 | 30.9 | 48 | 9.5 | 37.1 | 16 | 62.4 | 88 | 26.5 | 40.5 | 28.1 | 28.7 | 31.6 | 24.8 |  |
| **95% CIs** | 84.9–93.3 | 24.3–37.5 | 41.4–54.6 | 5.6–13.4 | 30.6–43.6 | 11-21 | 56.0–68.8 | 84.2–91.8 | 20.7–32.3 | 33.9–47.1 | 22.2–34.0 | 22.8–34.6 | 25.5–37.7 | 19.1–30.5 |  |
| **N blf** | 187 | 13 | 27 | 26 | 15 | 6 | 43 | 227 | 31 | 5 | 15 | 11 | 22 | 6 |  |
| **% blf** | 88.6 | 6.8 | 12.2 | 11.8 | 7 | 2.9 | 19.5 | 82.5 | 13.9 | 2.3 | 6.8 | 4.9 | 9.8 | 2.8 |  |
| **95% CIs** | 84.3–92.9 | 3.2–10.4 | 7.9–16.5 | 7.5–16.1 | 3.6–10.4 | 0.6–5.2 | 14.3–24.7 | 78–87 | 9.4–18.4 | 0.3–4.3 | 3.5–10.1 | 2.1–7.7 | 5.9–13.7 | 0.6–5.0 |  |
| **% blf inhibition** | ̶ | 92.2 | 86.2 | 86.4 | 92.1 | 96.7 | 78 | ̶ | 81.2 | 97.2 | 90.8 | 94.1 | 86.7 | 96.6 |  |
| **N dead imm** | 6 | 189 | 171 | 219 | 209 | 202 | 213 | 11 | 127 | 166 | 198 | 209 | 141 | 210 |  |
| **% dead imm** | 2.8 | 99 | 77.4 | 99.5 | 98.1 | 98.1 | 96.4 | 4 | 57 | 77.2 | 89.6 | 93.7 | 62.7 | 96.3 |  |
| **95% CIs** | 0.6–5.0 | 97.6–100 | 71.9–82.9 | 98.6–100 | 96.3–99.9 | 96.2–100 | 93.9–98.9 | 1.7–6.3 | 50.5–63.5 | 71.6–82.8 | 85.6–93.6 | 90.5–96.9 | 56.4–69.0 | 93.8–98.8 |  |
| **% corrected dead imm** | ̶ | 99 | 76.7 | 99.5 | 98 | 98 | 96.3 | ̶ | 55.9 | 76.3 | 89.3 | 93.4 | 61.8 | 96.1 |  |
| **N dead 24 h** | 9 | 190 | 179 | 219 | 212 | 204 | 220 | 14 | 140 | 175 | 216 | 219 | 201 | 216 |  |
| **% dead 24 h** | 4.3 | 99.5 | 81 | 99.5 | 99.5 | 99 | 99.5 | 5.1 | 62.8 | 81.4 | 97.7 | 98.2 | 89.3 | 99.1 |  |
| **95% CIs** | 1.6–7.0 | 98.5–100 | 75.8–86.2 | 98.6–100 | 98.6–100 | 97.6–100 | 98.6–100 | 2.5–7.7 | 56.5–69.1 | 76.2–86.6 | 95.7–99.7 | 96.5–99.9 | 85.3–93.3 | 97.8–100 |  |
| **% corrected dead 24 h** | ̶ | 99.5 | 80.1 | 99.5 | 99.5 | 98.9 | 99.5 | ̶ | 60.4 | 80.4 | 97.6 | 98.1 | 88.6 | 99.1 |  |
| **N dead 48 h** | 10 | 190 | 182 | 219 | 212 | 204 | 220 | 14 | 142 | 177 | 217 | 220 | 207 | 216 |  |
| **% dead 48h** | 4.7 | 99.5 | 82.4 | 99.5 | 99.5 | 99 | 99.5 | 5.1 | 63.7 | 82.3 | 98.2 | 98.7 | 92 | 99.1 |  |
| **95% CIs** | 1.8–7.6 | 98.5–100 | 77.4–87.4 | 98.6–100 | 98.6–100 | 97.6–100 | 98.6–100 | 2.5–7.7 | 57.4–70.0 | 77.2–87.4 | 96.4–100 | 97.2–100 | 88.5–95.5 | 97.8–100 |  |
| **% corrected dead 48 h** | ̶ | 99.5 | 81.5 | 99.5 | 99.5 | 98.9 | 99.5 | ̶ | 61.4 | 81.3 | 98.1 | 98.6 | 91.5 | 99.1 |  |
| **N dead 72 h** | 11 | 190 | 183 | 219 | 212 | 204 | 221 | 14 | 142 | 179 | 217 | 220 | 207 | 216 |  |
| **% dead 72 h** | 5.2 | 99.5 | 82.8 | 99.5 | 99.5 | 99 | 100 | 5.1 | 63.7 | 83.3 | 98.2 | 98.7 | 92 | 99.1 |  |
| **95% CIs** | 2.2–8.2 | 98.5–100 | 77.8–87.8 | 98.6–100 | 98.6–100 | 97.6–100 | ̶ | 2.5–7.7 | 57.4–70.0 | 78.3–88.3 | 96.4–100 | 97.2–100 | 88.5–95.5 | 97.8–100 |  |
| **% corrected dead 72 h** | ̶ | 99.5 | 81.9 | 99.5 | 99.5 | 98.9 | 100 | ̶ | 61.4 | 82.4 | 98.1 | 98.6 | 91.5 | 99.1 |  |
